# Data‐Driven Materials Science for Energy‐Sustainable Applications

**DOI:** 10.1002/adma.74491

**Published:** 2026-08-17

**Authors:** Jacqueline M. Cole

**Affiliations:** ^1^ Ray Dolby Centre Cavendish Laboratory Department of Physics University of Cambridge Cambridge UK

**Keywords:** batteries, data extraction, magnetic materials, materials discovery, photovoltaics, sustainable manufacturing, thermoelectrics

## Abstract

Materials science is underpinned by structure‐property relationships that govern the function of a material. These relationships can be encoded into algorithms and integrated into machine‐learning models that enable the prediction of materials and their cognate properties. However, machine‐learning models are largely being trained on computed data owing to a worldwide shortage of real‐world (experimental) datasets. This review describes how to capture and collate experimental data from scientific literature using artificial‐intelligence (AI) methods to produce materials‐domain‐specific datasets or language models. Their application in AI‐driven enquiries that facilitate progress in energy‐sustainable materials science is then illustrated via six case studies that cover: training machine‐learning models, data‐driven materials discovery, optimizing manufacturing processes, mapping phases of materials, forecasting materials‐centric research trends, and classifying types of materials using automated prompt engineering. The future of materials‐domain‐specific datasets, language models, and decision‐making workflows using AI agents is then envisioned for the energy sector. The intrinsic challenges of accessing historical dark data in materials science are then described and contrasted with timely opportunities for leveraging massive amounts of experimental data from laboratories in going forwards; by exploiting electronic‐lab notebooks, high‐throughput experiments, and digital‐twin technologies. These opportunities are illustrated for energy‐sustainable materials science, especially the photovoltaic and battery industries.

## Graphical abstract

Data‐driven approaches powered by artificial intelligence are transforming materials discovery for energy sustainability. This review examines how auto‐generated high‐quality materials databases and domain‐specific language models accelerate research in photovoltaics, thermoelectrics, batteries and magnetic materials. Applications involve extraction of useful material properties, forecasting trends and data‐driven materials discovery. We map challenges in data accessibility and outline emerging opportunities to acquire data.

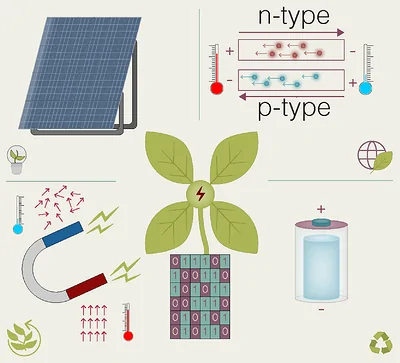

## 1 Introduction

The future of the Earth lies in the hands of the current generation of scientists and engineers. It is our responsibility to find technical solutions that practically meet the urgent global need for energy sustainability. Otherwise, climate change will continue inexorably to the destruction of our planet.

Many organizations around the world have advertised a strategy to become net zero by a stated target year, to help combat this global crisis. Yet, it is becoming increasingly clear that these strategies are woefully unrealistic unless new disruptive technologies come forward soon that offer a major step change in energy‐sustainable innovations which are of a nature that can be hard‐wired into the core operations of these organizations.

One of the most disruptive technologies of our time is artificial intelligence (AI). AI is already revolutionizing many industries, given its ability to automate repetitive tasks or predict outcomes without human input. ChatGPT offers a famous illustration of how AI has revolutionized everyday activities across the world [1]. Its revolutionary capabilities naturally beg the question as to whether or not AI could be used to discover technical solutions that lead to worldwide energy sustainability.

Such technical solutions will invariably require new materials for their implementation and application. AI has already been facilitating a ‘coming of age’ for the development of materials‐by‐design over the last few decades [2, 3, 4, 5]. Major political influences such as the Materials Genome Initiative [6] have helped to spearhead these advances. However, for all their mathematical brilliance, AI methods are useless in the absence of the right data for the job. Worse still, it is not trivial to secure such data, as will be shown in this paper. Accordingly, the digital revolution has deemed that ‘data are the new oil’ [7].

The employment of AI to deliver energy‐sustainable solutions also presents an intrinsic challenge. Thereby, less energy needs to be consumed in creating the AI solution than the energy that is saved by dispensing the solution. The danger of inflicting a false economy upon the planet is very real, given all the power‐hungry data centers that are being built around the world to meet the meteoric rise in global computing and data storage needs. The electricity consumption for data centers was 485 TWh in 2025, and this demand is projected to triple by 2030 for AI‐focused data centers, leading to data centers becoming responsible for 4% of the global energy demand by 2035 [8]. However, the news is not all bad, as long as AI and energy‐security developments are tightly linked. Thereby, AI‐automation is expected to digitally optimize production processes, including the operation of electricity grids themselves using digital twins, as will be exemplified in Section 4.3.7.3 of this review. Overall, contemporary AI‐automation use cases demonstrate the potential to save 3% of the global energy consumption by 2035 [8]. So, there is hope that energy supply and demand for AI could be met with a bit more innovation.

This perspective considers how a data‐driven materials‐science approach is starting to make headway in reaping the potential benefits of AI to help offset the global energy crisis. Given the *Advanced Materials Hall of Fame* theme of this article, the paper presents a personal perspective of the topic, describing general developments in the field while illustrating many of them using a contiguous series of detailed case studies from the energy sector. This detail is made possible by selecting studies that are particularly familiar to the author owing to their direct involvement in them. This detail should bring to a nuanced, added value to readers, especially those who may be interested in entering the field. Nonetheless, the author balances this familiarity‐based selection of examples by noting alongside these case studies relevant contributions from others that are especially seminal. Furthermore, the author takes care to envision the future of the topic by reviewing a broad set of myriad contributions from others.

This review paper begins by describing how data provisions are being developed in materials science for AI applications. With the recent major advances in computing power, a wealth of computational data can now be calculated *en masse* via high‐throughput computation [3, 4]. However, computed data need to be validated by experimental data [9]. Moreover, the ability to secure large volumes of experimental data is crucial to progress since experimental data relate directly to the real‐world challenges at hand [5].

This review therefore focuses on realizing the opportunity of experimental data. It begins by describing new ways to produce highly concentrated sources of collated experimental data for data‐driven materials science. To this end, one could argue that AI has helped to provision scientific data for itself because the two leading ways to capture large volumes of experimental data are via: (i) the AI‐enabled curation of materials databases by the automatic mining of scientific literature [10] and (ii) the tailoring of large language models (LLMs) for domain‐specific materials‐science applications [9].

The paper then shows how one can apply data‐analytical methods to these specially tailored databases or language models to deliver materials‐centered solutions for the sustainable energy sector. Six types of AI‐enabled data enquiries for energy‐sustainable materials‐science applications are illustrated through a series of case studies, each of which concerns a specific area of energy science. These enquiries concern the provision of: (1) auto‐generated materials databases; (2) data‐driven materials discovery; (3) data‐driven manufacturing and device physics; (4) data‐analytical phase mapping of materials; (5) data‐enabled forecasting of trends in materials science; (6) the classification of materials by fine‐tuning language models with question‐answering capabilities. These six types of AI‐driven data enquiry are summarized in Figure 1.

**FIGURE 1:**
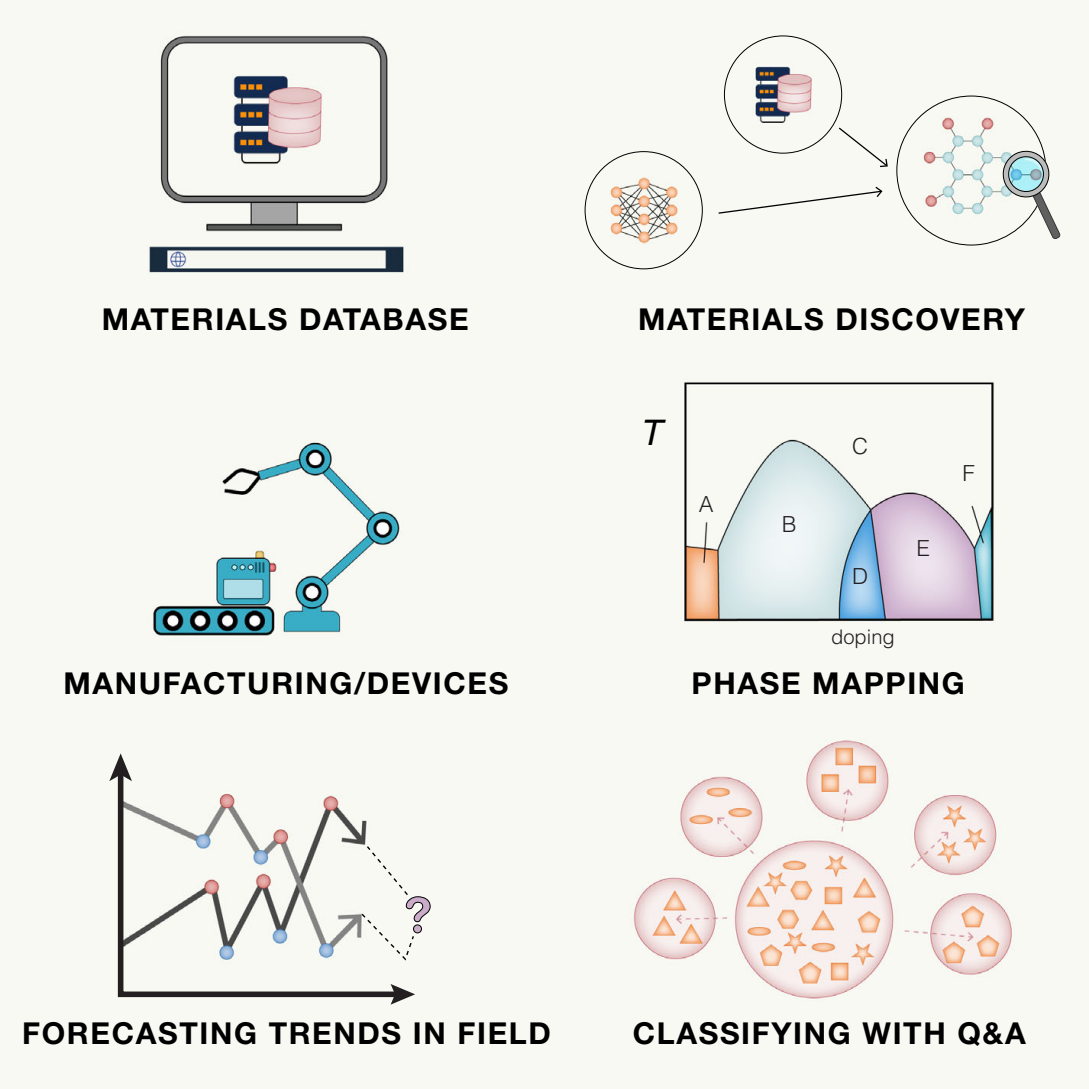
Six types of AI‐driven data enquiry that can facilitate progress in materials science.

This paper concludes with a future perspective on how AI methods ultimately need to be democratized to help offset climate change. A technical outlook with regards to the future development of language models is also presented. This includes an intrinsic need to find energy‐sustainable ways to train language models for materials‐science applications; otherwise, language models offer a false economy for their prospective use in energy science. The need for a global digital transformation in materials science is outlined, with special reference to the importance of accessing more high quality, diverse sets of experimental data. To this end, the challenges of dark data are considered, as are the prospective opportunities to harness materials‐science data in a way that provides suitable sets of experimental data to fuel the next generation of data‐driven materials science for the energy sector.

## 2 Provision of Experimental Data Using Artificial Intelligence

A limited number of large manually curated materials repositories of experimental data exist for scientific research. The most well‐known examples in materials science are probably crystallographic databases: the Cambridge Structural Database (CSD) [11], Inorganic Crystallographic Structural Database (ICSD) [12] and the Protein Data Bank (PDB) [13]. These databases have become established through the culmination of many lifetimes of human effort in manual data entry by dedicated editorial staff; their work has been supported by profits that have accrued from the commercialization of these databases and their associated properties. After many decades of editorial effort, such databases have grown to sizeable repositories; for example, the CSD now hosts data records about over 1.4 million crystal structures and their associated metadata [14]. These repositories are rare examples of large, structured, high‐quality, labeled, experimental datasets. As such, they have been employed in myriad scientific projects to solve all sorts of crystallographic challenges; one famous example being the use of the PDB in training the AI software, AlphaFold2 [15], that was recently the subject of a Nobel Prize [16].

However, not all types of materials crystallize; thus, they cannot be characterized by a crystal structure. Moreover, materials science is heavily informed by structure and property relationships, not just by structure. A different approach is therefore needed to define and collate experimental data about materials in terms of their structure *and* property characteristics. One alternative approach to these crystallographic databases is PubChem, which includes molecular structure and physico‐chemical and biological data on over 19 m small organic molecules [17]. PubChem was originally launched by the National Institute of Health and has continued to be developed and maintained by the National Center of Biotechnological Information. As a freely available product, its growth has been heavily facilitated by efforts from the wider research community. Nonetheless, such contributions of experimental data are manually curated. Moreover, PubChem has a natural predisposition toward biological properties, given its origins; and its corresponding applications are thus focused on drug discovery and related scientific pursuits.

Notwithstanding these important contributions to chemical and biological informatics, the increasingly pressing need for big data provisions across all areas of materials science renders manual‐database curation impractical. Furthermore, the rapidly evolving and interactive nature of AI means that we need a way to access data in an interactive medium to cope with the substantial rise in user demand for data with: (i) *high immediacy*; there is a distinct sense of “dial‐up data” needs; (ii) *dynamic profiles* to capture time‐dependent processes, for example, to create AI models based on historical trends; (iii) *standardized form*; so that they can be used to train AI architectures such as neural networks, or incorporated into data‐management frameworks such as knowledge graphs; (iv) *designer capabilities* so that they can be adapted, for example, to create encodings of correlated information which are known as *data representations*. Note that where such encodings pertain to structure‐property relationships, they are specifically known as *molecular representations*; such encodings lie at the heart of data‐driven materials discovery, especially in the realm of drug discovery [18].

In short, we need to turn the *status quo* of database management upside down: AI problems should not be built around an available database as they are largely now; rather, *data sources need to be custom‐built to solve a specific AI problem*.

The following subsections describe the two primary AI methods that are being employed to realize this paradigm shift.

### 2.1 AI‐Enabled Data‐Extraction Tools to Create Materials Databases

The essential challenge is to find a way that one can create dynamic, designer, structured sets of data for a given target challenge in materials science, in a timely fashion. This challenge can only be met if one can source large volumes of structure and property information with a high level of immediacy. Such data are available in the scientific literature, albeit in a highly fragmented form. These data are typically represented as text, image, or schematic information.

In principle, two adjacent fields of AI could help to serve AI‐enabled materials science. Thereby, natural language processing (NLP) and computer vision (CV) techniques can mine text or images from documents, with one important caveat: Such tools need to be specially equipped with ‘chemistry‐aware’ capabilities. In particular, chemical names and their cognate chemical labels need to be resolved within documents; this task is known as chemical named entity recognition (CNER). Additionally, their corresponding structure–property data within a given document need to be identified and interrelated. Olivetti and coworkers have presented an extensive review of the essential types of data‐mining methodologies that lie behind such ‘chemistry aware’ NLP and CV tools [10]. Their description also demonstrates that achieving such a task is far from trivial when it is required at a high‐throughput level, that is, when it needs to be automated, which is crucial for progressing modern‐day scientific research. Nonetheless, a range of software tools have been developed in order to rise to this challenge.

#### 2.1.1 ‘Chemistry‐Aware’ NLP Tools for High‐Throughput Text‐Mining

Olivetti et al. [10] reviewed a range of NLP tools that mine text for chemical applications. This review focuses on one of these tools, ChemDataExtractor, because it was the world's first open‐source ‘chemistry‐aware’ NLP toolkit for the high‐throughput text mining of scientific literature [19]. Its first‐mover advantage facilitated its global success as a platform technology for auto‐generating experimental materials databases. Its continued demand has led to subsequent developments of ChemDataExtractor v2.0 [20], v2.1 [21], v2.2 [22]. These version two options were developed in the wake of some of the first demonstrations that large‐language models (LLMs) could be used to successfully perform data‐extraction tasks from literature. Thereby, a chemical language model was developed and incorporated into ChemDataExtractor v2.1 to aid its CNER functionality [21]. A chemical language model is a special type of LLM that is pretrained exclusively on a long sequence of molecular representations that denote a form of chemical structure, for example, a long list of Simplified Molecular Input Line Entry System (SMILES) strings, as per the subject case [21].

Other hybrid software builds of ChemDataExtractor with LLM functionality include a property‐specific variation of ChemDataExtractor, known as BatteryDataExtractor [23]. In another example, others have recently incorporated ChemDataExtractor v2 and an LLM into a high‐throughput pipeline that forecasts the synthesizability of materials [24].

ChemDataExtractor has been used to successfully auto‐generate a range of large, structured materials databases of high quality for a variety of materials‐science applications (see Section 3.1 for details).

#### 2.1.2 ‘Chemistry‐Aware’ CV Tools for Image‐Mining

Companion open‐source tools to ChemDataExtractor that employ CV methods for mining complementary types of materials‐science data that exist within images or chemical‐schematic diagrams have also been developed by the same research group.

##### 2.1.2.1 Mining Images for Nanomaterials

ImageDataExtractor is an open‐source software tool for automatically extracting electron‐microscopy images from scientific documents and quantitatively analyzing them [25, 26]. Thereby, ImageDataExtractor detects particles within each image and quantifies their shape and size via a radial distribution function. Version 2.0 of this software [26] is particularly novel in its deep‐learning‐based instance‐segmentation method with Bayesian‐inference uncertainty quantification for particle detection. Amongst other benefits, this proposal‐free method enables segmentation in a single step, which avoids the error accumulation of proposal‐based (multistep) methods; the method also helps to resolve overlapping objects within images.

ImageDataExtractor has also been used to help automate the materials characterization of nanostructures. For example, it simulated the small‐angle scattering (SAS) data that trained a transformer model, SASformer [27], to predict the shape and size of nanoparticles ahead of the traditional first step of SAS data analysis. The latter involves presuming a specific type of structural topology for a nanomaterial (e.g., lamella, fractal, sphere, and disk) and then processing the SAS data on that basis; if this guess is incorrect, one has to return to the start; this SASformer‐based prediction method enabled by ImageDataExtractor thus affords a valuable short‐cut to SAS data analysis.

##### 2.1.2.2 Mining Chemical‐Schematic Diagrams

Companion tools to ChemDataExtractor include ChemSchematicResolver [28] and ReactionDataExtractor [29, 30].

ChemSchematicResolver [28] complements CNER‐based text‐mining procedures by decoding chemical schematic diagrams that depict molecules and their cognate chemical labels which are displayed exclusively within an image of a document. This tool extended the optical chemical structure recognition (OCSR) software, OSRA [31], with a python‐based approach and algorithms that enabled the resolution of unspecified chemical (R) groups in chemical schematic diagrams that are defined elsewhere in an image or in accompanying text. More modern tools have extended these capabilities much further. For example, the DECIMER.ai platform [32] first segments an image to isolate the chemical schematic diagrams therein; and then classifies each diagram using a neural network model to check that it is indeed a chemical schematic diagram; pending that to be the case, the diagram is converted into a SMILES string and automatically deposited into a database. The DECIMER platform has also been trained to handle hand‐drawn molecular‐images [33] which could be particularly useful for interpreting hand‐drawn or electronic laboratory notebooks (ELNs). Laboratory notebooks offer a rare type of data source in that they contain both positive and negative experimental results, which stand to be very valuable in future advances of data‐driven materials science, as will be discussed in Section 4.5.2.1.

ReactionDataExtractor [29, 30] complements these tools by extracting schematic diagrams of chemical reactions from figures in scientific documents and then classifies and decodes their image components into: reactants, intermediates, products, reaction conditions, and chemical labels. The decoding procedure includes converting the image components into text representations and chemical‐reaction graphs so that they are computer‐readable in readiness for the auto‐generation of chemical‐reaction databases. ReactionDataExtractor version 2 [30] embeds deep‐learning neural networks.

### 2.2 Language Models for Extracting Data in Materials Science

The emergence of LLMs has opened up new opportunities for all sorts of tasks, including the automated extraction of data from the literature. Indeed, automated text extraction was one of the first applications of foundation LLMs, as was particularly highlighted by the Bidirectional Encoder Representations for Transformers (BERT) architectures released by Google [34].

The open‐source nature of these BERT architectures soon led to the emergence of domain‐specific language models for materials‐science applications. Thereby, three independent research groups almost simultaneously demonstrated that a large volume of scientific literature on a given materials‐science topic could be collated into a large corpus of text and thence used to train an LLM that is geared for that specific scientific domain; in turn, it was shown that such an LLM could be fine‐tuned to perform downstream tasks for various applications that suit the materials‐science topic at hand. The first three corresponding materials‐domain‐specific BERT models were all published within almost a month of each other: MatSciBERT [35], MatBERT [36], and BatteryBERT [37]. The first two of these LLMs pre‐trained a BERT model for all materials science, while the BatteryBERT model pre‐trained an LLM specifically for the battery materials domain.

The Cole group has since published an entire family of property‐specific BERT models for the energy sector, that have been tailored for the specific materials‐science domain at hand: BatteryBERT [37], OpticalBERT [38], OE‐BERT (optoelectronics) [39], PVBERT (photovoltaics) [40], TE‐BERT (thermoelectrics) [41], MechBERT (mechanical engineering) [42], PhysicalSciencesBERT [43] and PhotocatalysisBERT [43]. These studies consistently showed that relatively small materials‐domain‐specific LLMs yield much better performance than much larger LLMs which have been pre‐trained on general English text. The data and performance specifications of these BERT models and those of the two wider materials‐domain MatBERT and MatSciBERT models are given in Figure 2.

**FIGURE 2:**
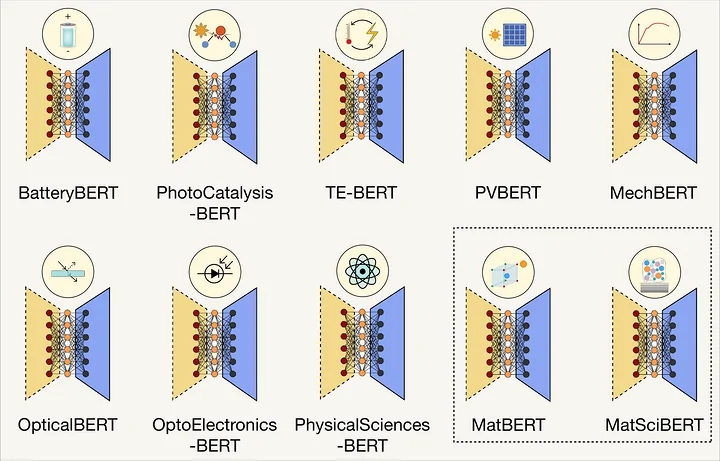
Domain‐specific BERT models developed for various fields of materials science. Each model is trained using a corpus of knowledge about a specific domain for: batteries (BatteryBERT), photocatalytic water splitting (PhotoCatalysis‐BERT), thermoelectrics (TE‐BERT), photovoltaics (PVBERT), mechanical properties (MechBERT), optical materials (OpticalBERT), optoelectronics (OptoElectronics‐BERT), and the broader physical sciences (PhysicalSciences‐BERT). MatBERT and MatSciBERT (dashed box) are language models that aim to cover the entire range of the materials sciences. All models share the BERT‐base transformer architecture (110 M parameters) but differ in corpus size, vocabulary and tokenization strategy. These differences complicate direct comparison of data‐to‐parameter ratios, since custom domain tokenizers produce fewer tokens (subwords) from the same corpus than the default WordPiece tokenizer.

Notwithstanding the underpinning technical importance of these domain‐specific LLM developments, it is important to note that their pre‐training requirements present considerable data and computational burdens, even for a foundation LLM that is very modest in size, such as the BERT‐base model whose architecture is defined by 110 M parameters.

Regarding the data challenge, one might ideally want to train an LLM with a data: parameter ratio of at least 10:1, as a basic statistical rule‐of‐thumb. Given that the natural training data for an LLM are text, a datum generally represents a ‘token’, which typically denotes any word, number or punctuation mark within a given corpus. The pre‐training of an LLM therefore requires a large textual corpus which contains at least 1.1 billion tokens. If the amount of data is too limited to serve this statistical measure, one might still succeed in pre‐training an LLM by optimizing the model over a commensurately larger number of training epochs; although this procedure naturally adds to the computational demands of pre‐training an LLM.

Regarding the wider computational challenges, it is helpful to consider the various ways that one can train an LLM. One may attempt to train a foundation LLM from scratch, whereby all the weights of its neural network are randomly reinitialized. Alternatively, one might train a foundation LLM from its starting weights, that is, those that exist in a ‘vanilla’ LLM prior to being exposed to textual input on a specific domain of interest. Cole and coworkers explored the relative merits of both options for pre‐training materials‐domain‐specific LLMs with BERT architectures, and concluded that there is generally no discernible benefit from training a foundation LLM from scratch [37, 38], albeit there can be exceptions [43].

Nonetheless, pre‐training an LLM from its initial weights still requires a substantial level of supercomputing resources. This resource requirement presents a serious problem because it directly undermines the inherent goal of energy sustainability [44], which is central to the scope of this review, and of course to the global energy crisis.

Recent developments in domain‐adaptive pre‐training (DAPT) methods [39, 45] can help to offset some of these energy requirements. Looking further ahead, knowledge‐distillation methods [46] offer considerable promise for their strong prospects in realizing a step‐change in energy savings. An extension of these methods from model‐to‐model mappings to data‐to‐model mappings empowers the use of small‐language models (SLMs) for materials science by essentially placing the ‘power of knowledge’ outside a language model; see Section 4.2.1 for further details.

This review focuses on domain‐specific BERT models for data extraction. Partly, this is to manage a contained discussion; indeed, a more comprehensive review is available on this topic [9]. Partly, this is because a community‐led development of BERT models has arisen from their open‐source nature [47], and because the BERT‐base architecture is sufficiently small that BERT models can be trained on widely available computing resources without an unbearable energy cost. BERT models can also be run off‐line, which can be crucial for meeting certain security needs. Additionally, BERT architectures offer better contextual understanding than other language‐model architectures, which makes them particularly well suited to tackling CNER tasks; this is a key consideration when extracting data for materials science, as was discussed in Section 2.1.1.

In stark contrast, while general purpose LLM architectures, such as Generative Pre‐trained Transformers (GPTs), have also been employed to perform data‐extraction tasks for materials‐science applications [48, 49, 50, 51], they contain domain‐specific information within an extremely large knowledge base; as such, they are not domain‐specific per se. Their performance is thus somewhat limited without significant human intervention, especially when being tasked to find intimate domain‐specific details about a material [43]. That said, prompt engineering can alleviate some of this human intervention to automate a considerable part of the data‐extraction process with good results [51]. Nevertheless, the use of most GPTs incurs a black‐box operation with: a financial cost, a larger energy cost, a mandate that a user cedes their information to the cloud to operate GPT, which invokes a security leak of data and precludes off‐line operation, which could be important in remote fieldwork. The large size of GPTs also hinders their utility as AI agents for autonomous operations where SLMs that can be tailored for one specific operation will be far more efficient; see Sections 4.2 and 4.3 for further details.

## 3 AI‐Enabled Data Enquiries for Energy‐Sustainable Applications

Having explained how to provision ‘big‐data’ sources for the materials‐science domain, their utility can now be showcased. To this end, the following six subsections illustrate their applications to the sustainable‐energy sector via a series of case studies that depict the types of AI‐driven data enquiries shown in Figure 1. These have naturally been chosen from collaborations involving the author, given their high‐end knowledge and qualified know‐how that is associated with these works. However, there are of course many other examples that are equally merit worthy; a few are mentioned without detail where relevant, in the following subsections, to help guide the reader to details of other studies.

### 3.1 Open‐Source Repositories of Experimental Data for the Research Community

The provision of large, high‐quality, experimental materials databases is perhaps one of the most valuable contributions that ‘big‐data’ materials‐science sources can offer. This is because of their widespread applications as information sources for ‘data look up’, data analytics, or machine‐learning (ML) applications.

In particular, ML, statistical inference, or their combination can be used to solve classification or regression problems in materials science, once their associated models have been trained, tested, or validated on domain‐specific data. Yet, such models are simply redundant without access to relevant data.

Large datasets of experimental data are particularly valuable for training models across a materials and property landscape. Thereby, information sources that contain sufficiently numerous and diverse data records can be representative of the entire population of data such that both in‐sample and out‐of‐sample predictions from associated ML models are viable. Figure 3a illustrates this point. The provision of balanced datasets of experimental data is important for similar reasons; Figure 3b illustrates how these points mitigate the over‐ or under‐fitting of data models [52]. Likewise, the influence of statistical errors caused by implicit data effects such as leverage needs to be considered (see Figure 3c).

**FIGURE 3:**
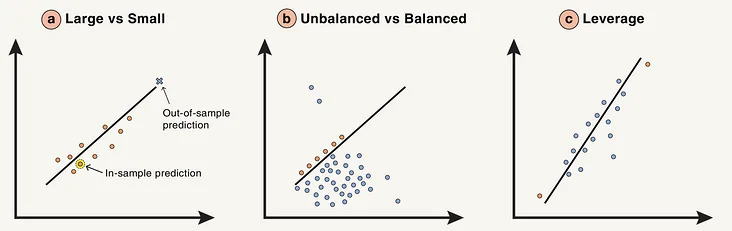
Common types of data that need to be considered when using data in ML modeling. (a) In‐sample data (orange) have been used to train or test an ML model prediction, while out‐of‐sample data (blue) are those that are used to evaluate the extent to which an ML model may be generalizable to all data [52]. (b) A balanced dataset (orange) is a collection of data that evenly spans a representative region of data that is used to train an ML model; an unbalanced dataset (blue) would be uneven within such a data region, resulting in bias toward to regions that are populated with more data; this bias could comprise the veracity of an ML model if such data were used to train or test it. (c) Statistical leverage refers to the extent by which the explanatory variable (x) of an individual data point within a dataset lies away from its sample mean value. A datum with particularly high statistical leverage (orange) lies at the extremities of a data range; the statistical modeling of a trend across such a data range will be disproportionally more influenced by these extreme data points, as they ‘anchor’ the boundaries of the model that is created to describe the data trend. Statistical leverage is more pronounced when the associated ‘anchor’ (x,y)‐data points do not align well with the statistical trend that is described by the rest of the sample data; thus, ML models derived from data with high statistical leverage carry a bias toward their values.

Experimental data are also important because they not only carry statistical error to aid uncertainty quantification but are also directly relatable to the real‐world applications for which they are being used.

Figure 4 displays a range of large, structured, domain‐specific materials repositories of experimental data that have been mined from scientific literature using the chemistry‐aware NLP tool, ChemDataExtractor. These serve as publicly available resources for the global research community [22, 53, 54, 55, 56, 57, 58, 59, 60, 61, 62, 63].

**FIGURE 4:**
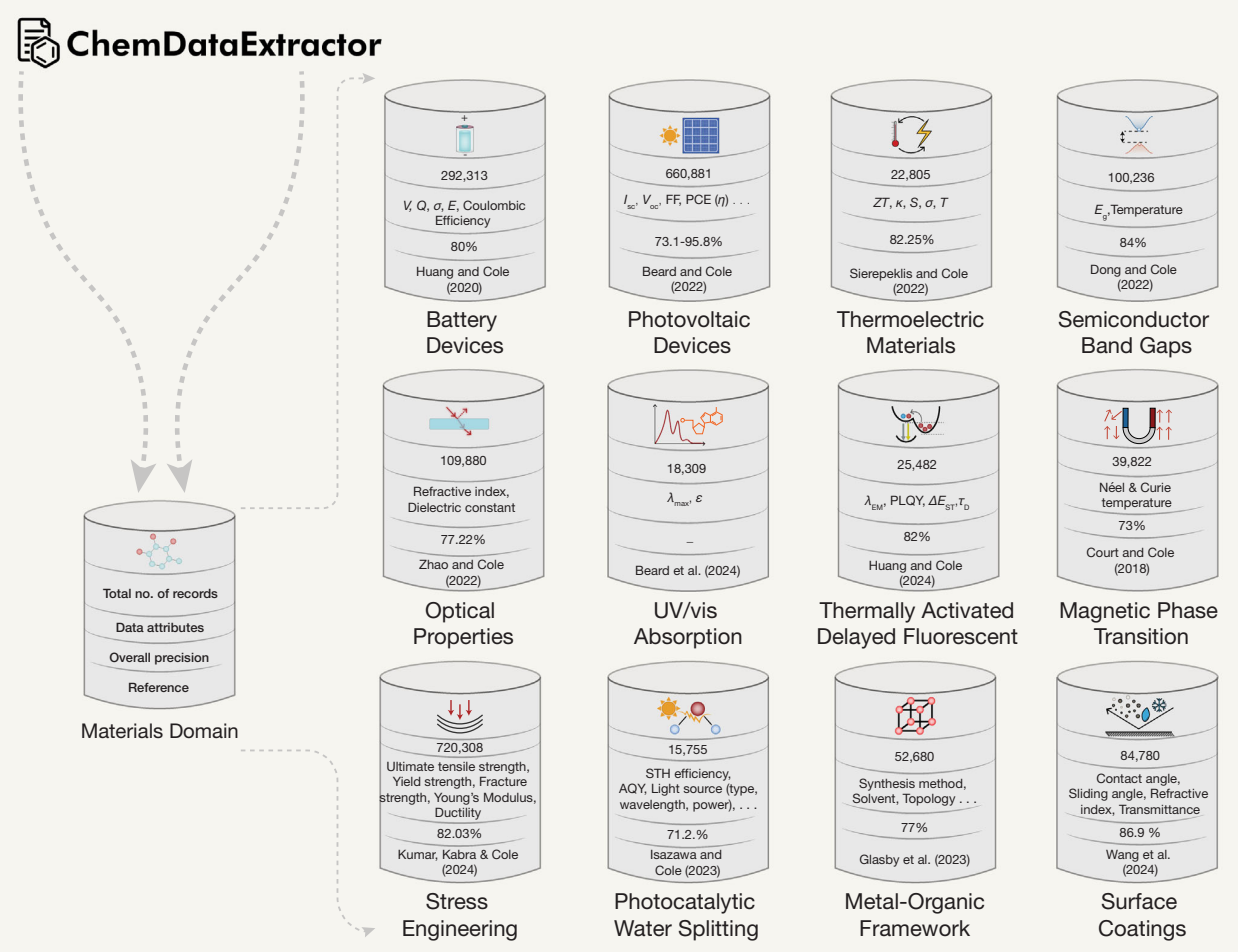
A range of large, structured, high‐quality, domain‐specific materials repositories of experimental data that have been auto‐generated by mining the scientific literature using the chemistry‐aware NLP tool, ChemDataExtractor [22, 53, 54, 55, 56, 57, 58, 59, 60, 61, 62, 63].

#### 3.1.1 Quality Control of Auto‐Generated Experimental Materials Databases

High‐quality materials databases are naturally essential for ‘data look‐up’ applications, since these needs are deterministic; notwithstanding that database auto‐generation tools, such as ChemDataExtractor, collect the Document Object Identifier (DOI) of the paper from which each data record was acquired so that a database user can manually verify any data record.

High‐quality materials‐database sources are also important for ML and statistical‐inference applications, although there is some tolerance in this regard. This is because data‐analytical methods tend to filter out statistical outliers by their inherent pattern‐seeking nature. Thus, ML and statistical‐inference models can withstand a certain amount of rogue data within a given data source; although their level of robustness to such inaccuracies will depend upon the statistical leverage of any rogue data point (Figure 3c), as well as the characteristics of the overall data source (e.g., the extent by which the underpinning training data are balanced; see Figure 3b), and the inherent quality needs of the materials‐centered problem at hand.

For example, a less robust materials database might be one that is highly complex, in the sense that it contains, say, the chemical name of a given material and many of its cognate properties that exhibit low correlations to each other (i.e., low *R* ^2^). In contrast, a materials database whose records simply depict the chemical structure and a cognate property that are correlated by a well‐established, straightforward linear structure‐property relationship will tend to be very robust, because any data outliers would be simple to diagnose.

A related point is the need to consider the consequences of an incorrect data‐analytical prediction owing to the use of inadequate training or testing data in the construction of the predictive model. For example, one can imagine that an ML model which incorrectly predicts a cancer diagnosis owing to rogue training data has greater consequences than an ML model that incorrectly predicts the nicest color of paint to decorate a bathroom. Data‐cleaning tools are now available that remove statistically evident rogue data from initial ML prediction models to help mitigate such consequences [64], although such automated data‐cleaning options may remove ostensible data outliers that could be the truly novel data that bucks the trend to provide a key innovation or discovery.

Databases that have been auto‐generated by tools, such as ChemDataExtractor, have been technically evaluated using precision, recall, and F1‐score metrics, whereby:

(1) $$\mathrm{Precision}=\frac{\mathrm{TP}}{\left(\mathrm{TP}+\mathrm{FP}\right)}$$

(2) $$\mathrm{Recall}=\frac{\mathrm{TP}}{\left(\mathrm{TP}+\mathrm{FN}\right)}$$

(3) $$F1=\frac{2\left(Precision\right)\left(Recall\right)}{\left(Precision+Recall\right)}$$

where counts are categorized as true positive (TP), false positive (FP), or false negative (FN).

In the context of automated data‐extraction capabilities, precision reflects the propensity by which extracted data that are included in a given database represent the intended data. Meanwhile, recall refers to the extent to which a data‐extraction tool can capture all the implicitly available target data from its textual source input.

Precision and recall often end up competing against each other because a focus on extracting data correctly (leading to high precision) is often accompanied by the setting of filters that block unwanted data; the more stringent that filters become, the higher the prospective data quality but the loss in correct data through such filters (together with the unwanted data), which leads directly to low recall. In contrast, high recall is often associated with very relaxed acceptance criteria for data‐extraction processes, which leads to low precision scores. Nonetheless, a high F1 score, which reflects high precision and high recall metrics, can be achieved through the careful balancing of data‐acceptance criteria during data extraction and the use of high‐quality data‐extraction algorithms; some of which may have the capability to be trained; see, for example, the recently published semi‐supervised Snowball v2 algorithm [65]. Data‐cleaning tools for materials science have also been constructed to help improve F1‐score metrics [66].

It should also be borne in mind that auto‐generating materials databases which contain many properties will tend to display lower overall evaluation metrics; this is a natural consequence of the greater challenge of extracting more types of property data per database. Overall, precision, recall, and F1 scores offer useful indicators of the quality of an auto‐generated materials database, with reference to the aforementioned caveats.

### 3.2 Data‐Driven Materials Discovery

Materials databases are increasingly being applied to ‘data‐driven design‐to‐device’ pipelines for materials discovery [5, 67]. Such pipelines aim to replace the traditional ‘trial‐and‐error’ efforts in chemical synthesis to discover new materials for a given application. ‘Big data’ approaches enable the wealth of the chemical knowledge that has been gathered historically to be used to find patterns in data that represent structure–property relationships that pertain to a given target application. Such relationships can then be employed to design and predict materials for a target application. Only the lead candidates from such materials predictions then need to be experimentally validated, therefore yielding a much more targeted chemical synthesis and device manufacturing and testing supply chains compared to ‘trial‐and‐error’ efforts. This is stimulating a paradigm shift which is very much in the spirit of the Materials Genome Initiative (MGI) [6] that was launched in the US under the Obama government.

The MGI recognizes a need to overcome the major materials bottleneck that is stifling industrial progress. In essence, it generally takes far too long to find a new material that befits a technological innovation. The MGI aims to accelerate the ‘molecule‐to‐market’ pipeline in industry, from an average 20 to 5 year timeframe. Data‐driven design‐to‐device pipelines present the ideal vehicle to realize this goal. Given that data lie at the front end of such pipelines, the provision of materials databases, which have been tailored to the needs of the materials challenge at hand, is pivotal to the success of the MGI.

Figure 5a illustrates a data‐driven design‐to‐device pipeline for discovering new light harvesters for photovoltaic (PV) applications, which is the subject of the case study that will now be discussed [68]. The type of PV technology of concern here pertains to dye‐sensitized solar cells (DSCs) [69]. However, light‐harvesting chromophores for all types of PV device technology share an underpinning need for a strong optical‐absorption characteristic.

**FIGURE 5:**
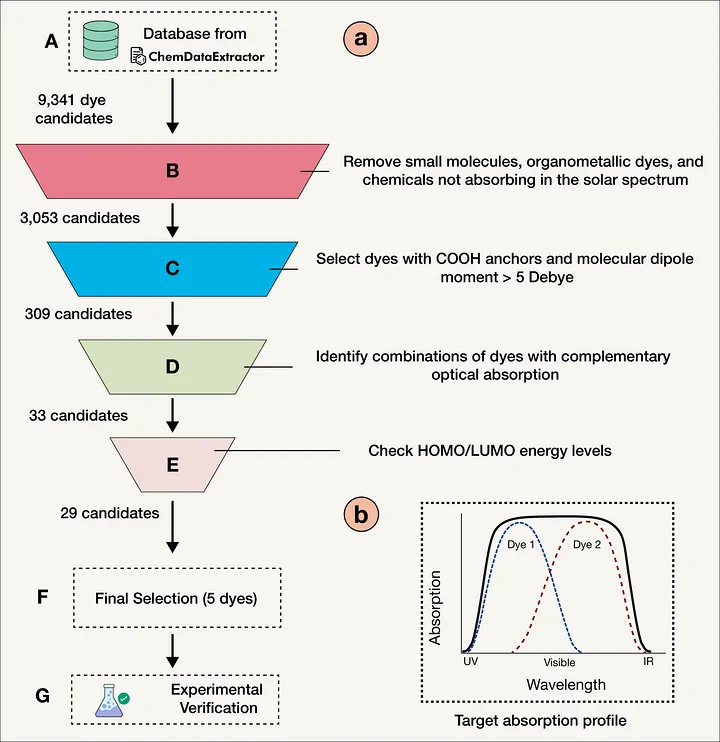
(a) The ‘design‐to‐device’ decision‐making workflow for the data‐driven materials discovery of new light‐harvesting molecules for photovoltaic applications. (b) An illustration of how two dye molecules with complementary optical‐absorption characteristics can be used together to create panchromatic optical absorption in a solar cell. (a) was adapted from Cooper et al. [68]. (b) was adapted from Cole et al. [70].

In essence, one wants a light‐harvesting material that captures all photons that are emitted from the sun. The ideal scenario would then be a molecule whose optical absorption spectrum maps exactly onto the solar‐emission spectrum. While there is no known molecule that can achieve such panchromatic absorption, it is possible, in principle, to realize this extent of optical absorption by combining the complementary absorption spectral characteristics of several chemical molecules within a single PV device; for example, employing a pair of light‐harvesting molecules in a DSC, one of which absorbs in the red, the other in the blue; such that the convolution of their individual absorption spectra results in a panchromatic absorption signature (see Figure 5). This strategy is commonly used in practical PV device design; it is known as dye co‐sensitization [70]. We therefore translated this into a data‐driven design‐to‐device strategy.

The data challenge therefore became one of creating a database of chemical molecules and their cognate maximum wavelength of absorption, *λ*_max_, and, where present, their molecular extinction coefficient, *ε*; and then coupling this with an algorithmic search engine that asked a carefully crafted and sequenced set of practical questions about the suitability of a chemical molecule for the target application. The answer to each question determined whether or not a given chemical would be short‐listed to the next stage of further enquiry. The fine details of the decision‐making surrounding the choice of each question and answer in this case study are presented in the boxed area for the interested reader (Box 1).

Questions continued to be asked until the short‐list retained only a handful of chemicals. Such chemicals that satisfied every question in the series of enquiries were deemed to be lead candidate dye molecules for DSC light‐harvesting applications. The lead candidate materials were taken forward for experimental validation, via the chemical synthesis of each dye and their subsequent incorporation into a PV device through DSC device fabrication and testing.

One of the greatest aspects of this data‐driven ‘design‐to‐device’ approach is that each predicted lead candidate dye is known to be “synthesizable”. This is because the data that led to the materials prediction in this pipeline arose from a database whose contents were, in turn, sourced from the scientific literature. So, each lead candidate dye had been synthesized previously, albeit for completely different reasons; motivations were mostly associated with synthetic curiosity. Moreover, each record in the materials database is tagged with the DOI of the paper from which it was sourced. Thus, we were able to contact the corresponding author of the original paper that synthesized and characterized each lead candidate chemical and its molecular absorption properties (*λ*_max_, *ε*); all such authors agreed to send samples. Their synthetic expertise was leveraged through a collaboration whereby they remade and sent samples to us for DSC device fabrication and testing that we performed. This highly directed access to specialist synthetic chemists is important because chemists tend to become expert in a particular area of synthetic chemistry; few are “generalists”. Accordingly, one needs to match a material prediction with a chemist who possesses appropriate synthetic expertise.

Indeed, the key ‘discovery’ paper that pertains to this study contains 18 authors [68], most of whom are the synthetic chemists who repurposed their expertise to provide us with samples of our lead candidate predictions. We experimentally validated these light‐harvesting dyes by incorporating them into co‐sensitized DSC devices and testing their PV performance characteristics. The power conversion efficiency (PCE) of one of these co‐sensitized DSCs was spectacular: 92% of the PCE of the industrial standard: a ruthenium‐based organometallic dye, known as N719 [71]. At this stage, it is helpful to remember that the first question in our aforementioned short‐listing process retained only organic dyes on the basis of environmental considerations. So, this result represented one of the world's highest‐performing pairs of organic dyes in a DSC device at the time of publication. This demonstrates the power of the data‐driven design‐to‐device approach to materials discovery.

On a more philosophical point, it is sometimes contended that this result does not represent ‘materials discovery’ but rather, the *re‐purposing of known materials*. This is true, to an extent. However, it is perhaps more apt to present such work as “materials discovery for a target application” since while the materials had been synthesized previously, they were not known for DSC applications. There is currently a prevailing tendency in the field of data‐driven materials discovery to only consider materials as being “discovered” if they have never been made before their prediction. This is perhaps symptomatic of the ‘hype’ or drive toward generative AI where “hypothetical” materials can be predicted for a target application. Their sequential discovery would naturally represent a truly new material. However, one could argue that it is equally, if not more, valid to *discover a new utility for a known material*, because it presents a more energy‐sustainable solution to materials discovery.

First, it can be synthesized by a known reaction mechanism, and so it can be remade without lots of ‘trial‐and‐error’ synthetic efforts, which would somewhat defeat the object of data‐driven materials discovery given one of its key goals is to overcome ‘trial‐and‐error’ synthetic approaches. Secondly, the a priori known synthetic efficiency associated with our “synthesizable” approach will result in less chemical waste being created during our experimental validation stage of materials discovery, as well as offer safer chemical practices given that a previously tested synthetic methodology will be employed. Thirdly, this material re‐purposing methodology aligns very well with the materials reuse aspects of the European Union's (EU) Critical Raw Material Act [72] and their associated materials sustainability goals.

This case study has presented a data‐driven design‐to‐device approach that uses experimental data as its source, and whose prediction strategy employs a series of questions to successively filter a large pool of material candidates to a few lead materials which are then experimentally tested. It is perhaps helpful to add that there are not yet very many examples of data‐driven materials discovery, although there are many examples of data‐driven materials prediction, that is, many material predictions that lack experimental validation. Moreover, such examples typically employ computational databases as their sources and use machine‐learning algorithms for materials prediction. One notable example of a study that uses virtual screening with machine‐learning and yet bucks the trend in the bottleneck of experimental validation is a data‐driven design‐to‐device pipeline that discovered new materials for organic light‐emitting diodes [73].

**Box 1. Paring down a materials database to a handful of lead candidate materials**

A lead material for a target application can be identified from a large materials database by applying a multistage filtering process. This process involves posing a carefully designed set sequence of questions about each material in the database that probe the relevance of their properties to the target application. The same set of questions is asked of each material at each stage of this filtering process. The selection and sequencing of such questions is designed in such a way as to pare down efficiently the large number of chemicals in the materials database to a handful of chemicals that can be taken forward for experimental validation. Thus, the initial questions in this set sequence should be of a nature that imposes a rough filter so that many chemicals are removed from the list of candidates early on; otherwise, shortlisting would take an unmanageable number of steps (questions). Accordingly, the number of materials being considered at each stage of the process tends to stack as an inverse pyramid of materials that funnels through to the bottom only a few chemicals that meet the criteria of all questions posed in their set sequence (see Figure 5).

It is important to understand that the exact nature of these questions, and thus the filtered set of candidate lead materials, are not unique. There are many other ways to successfully shortlist materials for a given functional application. Nonetheless, the ‘art’ of the questioning is paramount to the practical success of the process. In effect, this is a manual version of “prompt engineering” ‐ the same type of ‘art’ that is now being applied regularly to large language models to acquire information (see Sections 2.2 and 3.6 for more details).

Let's now illustrate the use of this prompt engineering ‘art’ by considering the exact questions that were asked in our case study on DSC dye discovery [68]. Thereby, we will demonstrate the rationale behind the choice of the questions and how we can classify their type of query. This case study involves paring down a materials database of just under 10,000 molecules to five lead candidate materials that were taken forward for experimental validation: chemical synthesis, materials characterization, and thence, device fabrication and testing.

A sequence of four questions was judiciously selected to identify these five lead candidates. The first question excluded metals from material candidates to comply with *environmental concerns* to afford a short‐list comprising organic materials exclusively. Only a third of materials from the parent materials database were permitted to pass through this filter.

The second question probed a key DSC *device requirement* of the target dye materials. The question effectively asks if each dye molecule possesses a carboxylate group. This is because the dye molecule needs to adsorb onto the surface of a titanium dioxide semiconductor to form the dye^…^TiO_2_ interface that constitutes the working electrode of the solar cell; and a carboxylate group within a dye molecule offers one of the strongest types of dye^…^TiO_2_ adsorption [74]. An order‐of‐magnitude drop in the remaining number of candidate dyes resulted from this second enquiry.

The third question filtered the remaining dyes according to an *algorithmic optimization* procedure. Thereby, an in‐house algorithm was built to identify pairs of dyes that absorb in the most mutually complementary parts of the visible region of the electromagnetic spectrum, *such that a pair of dyes will exhibit panchromatic optical absorption* when co‐sensitized in an overarching DSC device [70], *e.g*., one dye that absorbs red light and another that absorbs blue. Another order‐of‐magnitude drop in the number of dye candidates was afforded via this filter.

The fourth question checked if the *molecular energetics* of the remaining dye candidates can align with the energy band that is a priori regulated by DSC device characteristics. This removed four dye candidates, leaving 29 possible options of DSC light‐harvesting dyes.

A manual check was then performed on these dye options that finally filtered on the practical grounds of their *novelty, synthesizability, and chemical availability*.

This led to the final five candidate materials that were experimentally validated, the result of which yielded a new pair of organic dyes which, once co‐sensitized and incorporated into a DSC device, yielded a 92% performance conversion efficiency relative to that of a DSC that had been sensitized by an industry‐standard organometallic dye [71]. This demonstrates the power of our approach.

Nonetheless, there are of course other ways to screen materials for data‐driven discovery. Popular methods include screening computationally‐generated materials on formation energies, or imposing a machine‐learning screening method. Meanwhile, prompt engineering has recently become a very popular option, albeit via LLMs rather than from static materials databases.

### 3.3 Auto‐Generating Materials‐Device Data for Sustainable Manufacturing

‘Chemistry‐aware’ text‐mining tools such as ChemDataExtractor specifically target the extraction of chemical names from documents together with their associated data; such chemical names are akin to a ‘primary identifier’ in the sense of traditional database semantic terminology. Yet, such tools are completely ambivalent to the type of *associated* data that they extract. Thus, ChemDataExtractor can be employed beyond its aforementioned use in auto‐generating materials databases that couple chemical and molecular property information.

For example, ChemDataExtractor has been employed to auto‐extract chemical names and their cognate macroscopic device data from scientific literature. A case in point concerns two large materials‐device databases for the PV field: one for DSCs and the other for perovskite solar cells (PSCs) [54]. The DSC database contains nearly ½ million data records (475K), which is considerably larger than the PSC database that comprises 186K data records. This stands to reason because many more DSC data are available in the literature, as DSCs represent a much more mature field of PV technology than PSCs.

Both DSC and PSC databases feature data about the four main device performance metrics that are typically reported together in papers: short‐circuit current density, *J*_sc_; open‐circuit voltage, *V*_oc_; fill factor, FF; and power conversion efficiency, PCE. Figure 6 presents these data in terms of their statistical distributions. These histograms illustrate a range of device features that are commonly observed in statistical distributions of experimental data. The quality control of these data was supported by performing a range of standardization, statistical imputation and verification tasks which are described in the next sub‐section.

**FIGURE 6:**
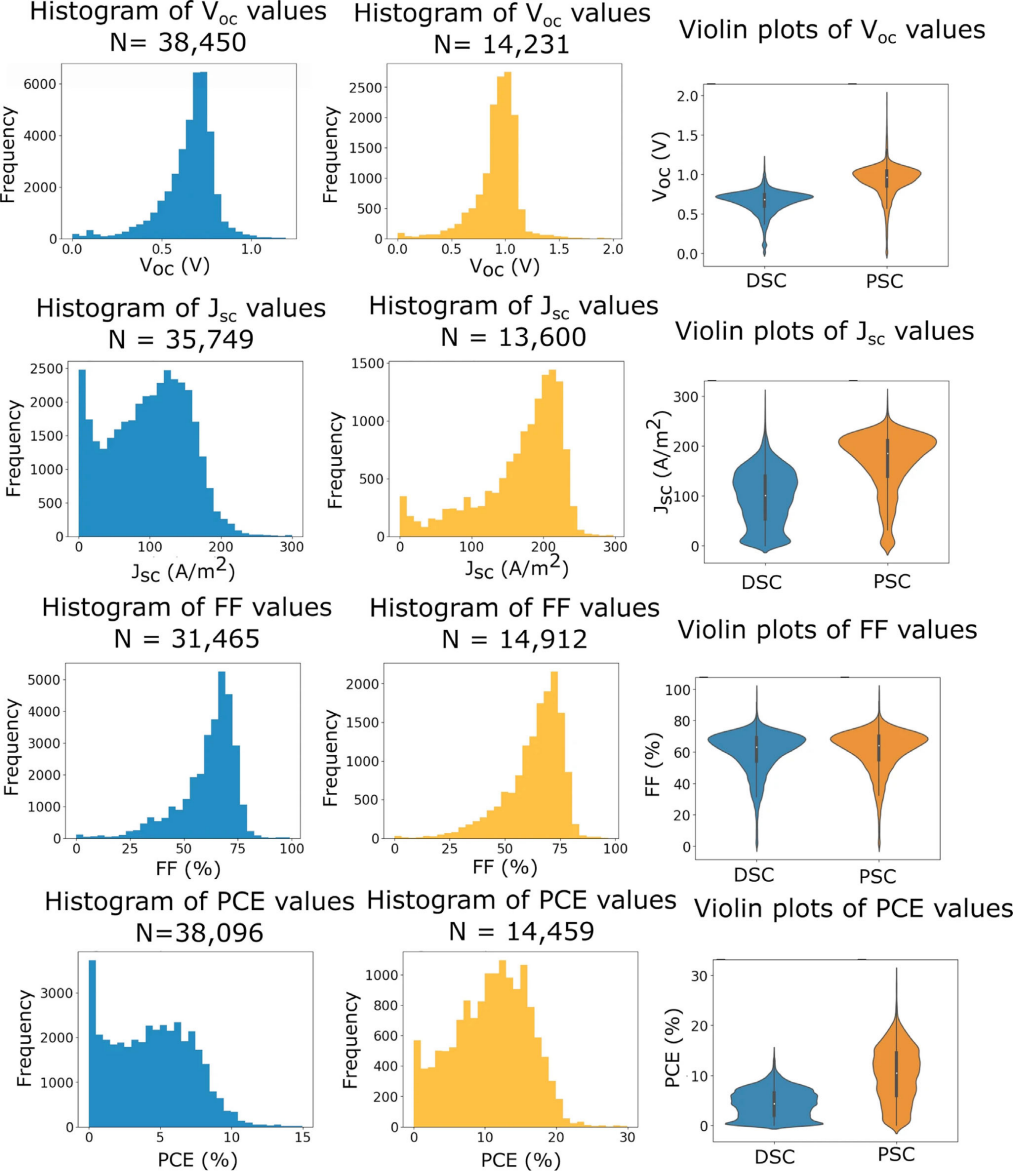
Statistical distributions of device characteristics in dye‐sensitized solar cell (DSC) and perovskite solar cell (PSC) databases. Histograms of open‐circuit voltage, *V*_oc_; short‐circuit current density, *J*_sc_; fill factor, FF; and power conversion efficiency, PCE, for (left) DSC and (middle) PSC databases; (right) violin plots of these four device properties for the DSC and PSC databases. Adapted from Beard & Cole [54].

#### 3.3.1 Quality Control of Device Data: Standardization, Statistical Imputation, and Verification

##### 3.3.1.1 Standardization

The reliability of many PCE values that have been reported in the PV literature has been challenged, particularly PCE values that lie in older reports. This is with good reason because the PV field went through a phase of unregulated reporting in terms of its device performance metrics. The problem was so acute that the research community has partnered with publishers to develop regulations on the reporting of PV device performance metrics [75]. This issue predominantly affected the older PCE values in the DSC device database presented herein. Fortunately, the frequency of erroneous claims was damped once these regulations had been implemented by key publishing houses. PCE values in the PSC device database have been better safeguarded because PSC technology emerged later, when regulations were already in place.

Nevertheless, one can ‘fix’ these DSC device reporting errors to an extent by calculating the ratio of the PCE for a DSC containing a new light‐harvesting dye against that of a DSC containing the industrial‐reference light‐harvesting dye that is reported in the same paper as the new dye being published. This affords a relative PCE value, according to the equation:

(4) $$\mathrm{PCE}\left(\mathrm{relative}\right)=\frac{\mathrm{PCE}\left(\mathrm{new}\;\mathrm{light}\;-\;\mathrm{harvesting}\;\mathrm{dye}\right)}{\mathrm{PCE}\left(\mathrm{reference}\;\mathrm{light}\;-\;\mathrm{harvesting}\;\mathrm{dye}\right)}$$

This ‘fix’ proved to be fruitful in ensuring good quality control of the DSC device database because PCE values from DSCs containing industrial‐reference light‐harvesting dyes are frequently reported alongside those that contain a newly reported light‐harvesting dye. To put this into perspective, 19 318 PCE values were extracted on the industrial‐reference DSC dye, N719 [71]. Moreover, this PCE(relative) ratio metric tends to be reliable because the PCE values for the pair of DSC devices containing either the new or reference light‐harvesting dyes have typically been measured by the same researcher, who has fabricated the devices in the same batch, using the same solar simulator on the same day in the same laboratory.

##### 3.3.1.2 Statistical Imputation

The DSC and PSC databases also contain other PV device characteristics where they were reported in, and extracted from, the sourced paper. Such characteristics include the solar irradiance exhibited by the PV device.

An interesting feature of this case study on auto‐generated PV‐device databases is that their aforementioned four primary PV‐device performance metrics are all interrelated to the solar irradiance via the equation:

(5) $$\mathrm{Solar}\;\mathrm{Irradiance}=\frac{\left(V_{\mathrm{oc}}\times J_{\mathrm{sc}}\times \mathrm{FF}\right)}{\mathrm{PCE}}$$

Accordingly, this equation provided an opportunity to calculate the value of any one of its variables where the data‐extraction process had failed to retrieve it and yet had successfully extracted the other four PV‐device performance metrics. Having calculated these missing values, they were substituted into the relevant empty data records of the corresponding PV‐device database, that is, we performed statistical imputation. In principle, any of these device characteristics could be calculated. However, the missing values were typically associated with the solar irradiance, since this device characteristic is the least frequently reported in the literature out of the five equation variables.

##### 3.3.1.3 Verification

It was also possible to use this equation for quality control in terms of verifying data entries, in cases where values for all five device characteristics are present in these databases; since the equation should naturally balance once all five values have been substituted therein.

Meanwhile, the precision of the data‐extraction process was calculated in terms of that which correctly afforded the light‐harvesting chromophores as well as the four aforementioned primary device performance metrics: *J*_SC_, *V*_OC_, FF, and PCE. This precision metric was found to be 84% for both the DSC and PSC materials device databases.

#### 3.3.2 Device Metrology Data

These two PV device databases also captured a range of “data metrology” data, wheresoever ChemDataExtractor could identify them for data extraction; such information is typically present in the “experimental methods” sections of scientific papers. These data include the active area of a solar cell being tested, the thickness of the semiconductor that supports the light‐harvesting chromophore, and the type of solar simulator that was used to measure the device characteristics of a given solar cell. The performance of a PV device can be heavily impacted by the nature of such “device metrology” data.

Accordingly, the provision of such data stands to be very useful for the design engineering of future PV devices. Given that device manufacturing processes tend to develop in a trial‐and‐error fashion, a data‐driven approach to device design would be far less wasteful in resources, thus leading to more sustainable manufacturing processes. Indeed, other research groups have also used ChemDataExtractor to auto‐generate databases for the needs of manufacturing; see, for example, a repository of material and metrology data about self‐cleaning coatings [63].

#### 3.3.3 The Scale of Data: From Atomic to Device Information

We conclude this section with a final, but important, comparative note between experimental materials databases that have been created by NLP‐enabled literature mining and computationally‐generated materials repositories. As was mentioned earlier, NLP has an advantage over high‐throughput calculations in terms of its ability to auto‐generate large databases of experimental (real‐world) information. The use of ChemDataExtractor in auto‐extracting macroscopic device information via the case study presented in this section highlights another key advantage of ‘chemistry‐aware’ NLP tools, which is that they are agnostic to scale. In stark contrast, high‐throughput electronic‐structure calculations can only compute atomic‐level structure and their associated properties. Thus, tools such as ChemDataExtractor are far more versatile in their database generation capabilities.

### 3.4 Uncovering New Phases of Materials by Data‐Driven Mapping Phase Space

ChemDataExtractor has also been used to stitch together numerical property data about related chemicals from the scientific literature which map a graphical trend owing to the presence of underpinning structure‐property relationships. Such mappings can be used to identify such structure‐property relationships, or to discover new material structures or properties by extrapolating from the graphically mapped structure‐property data or interpolating between gaps in these data. This is perhaps best illustrated via a case study, which concerns the data‐driven mapping of phase space for magnetic materials.

Ferromagnetic materials are of particular interest for energy‐sustainable applications, given their capacity to afford magnetocaloric effects. Such effects are well‐known for their applications to magnetic refrigeration. For example, ferromagnetic materials that exhibit magnetocaloric effects at temperatures close to the liquefaction temperature of hydrogen (∼20 K at atmospheric pressure) have been predicted using ML methods in two studies, given their potential use as magnetic refrigerants to store hydrogen as an energy‐sustainable fuel [76, 77].

Incidentally, ChemDataExtractor was used to auto‐generate the materials database containing characteristic properties for magnetocaloric effects that was needed to perform the ML predictions in both of these studies; thus, showcasing another example of the use of ChemDataExtractor in providing open‐source repositories that contain structure‐property relationships for AI‐enabled data enquiry (cf. Section 3.1).

ChemDataExtractor was also employed at the front end, in the context of this case study, to auto‐generate a large database of chemical materials that report cognate Curie (*T*_C_) and Néel (*T*_N_) temperatures in the scientific literature; these property data were specifically suited because they pertain to ferromagnetic and antiferromagnetic phase transitions, respectively [60]. These phase‐transition temperatures are typically mapped out as a function of the stoichiometric chemical composition, x, of a given family of magnetic materials, that is, *f*(x,*T*). This phase‐space mapping helps researchers to identify and interpret the various magnetic phases that can exist within a class of materials. This is because magnetic phase transitions provide the boundary lines between areas of distinct forms of magnetism. Therefore, visual projections of these boundary lines plotted across this phase space can predict new magnetic phases of a given class of materials, wheresoever areas within these maps are found to be unchartered.

In principle, this sounds like a simple exercise. However, the magnetic research community typically report individual *T*_C_ and *T*_N_ values for a given material and composition, x, in a bespoke publication. The collective information, *f*(x,*T*), therefore becomes strewn across the scientific literature. As such, it is a substantial manual undertaking to source and then map phase space for a given family of magnetic materials; this heavily compromises the predictive utility of mapping magnetic phase‐space. Fortunately, tools such as ChemDataExtractor provide a way to automatically identify, extract and collate this highly fragmented type of data.

Figure 7 illustrates a case in point for the family of La_1‐x_Sr_x_MnO_3_ magnetic materials. Figure 7a maps its phase space using the collation of *T*_C_ and *T*_N_ data points for these materials that ChemDataExtractor had extracted from the scientific literature [78], which we connected in a dot‐to‐dot fashion. The resulting bounded areas that are produced in the map automatically reveal various structural phases of this family of materials.

**FIGURE 7:**
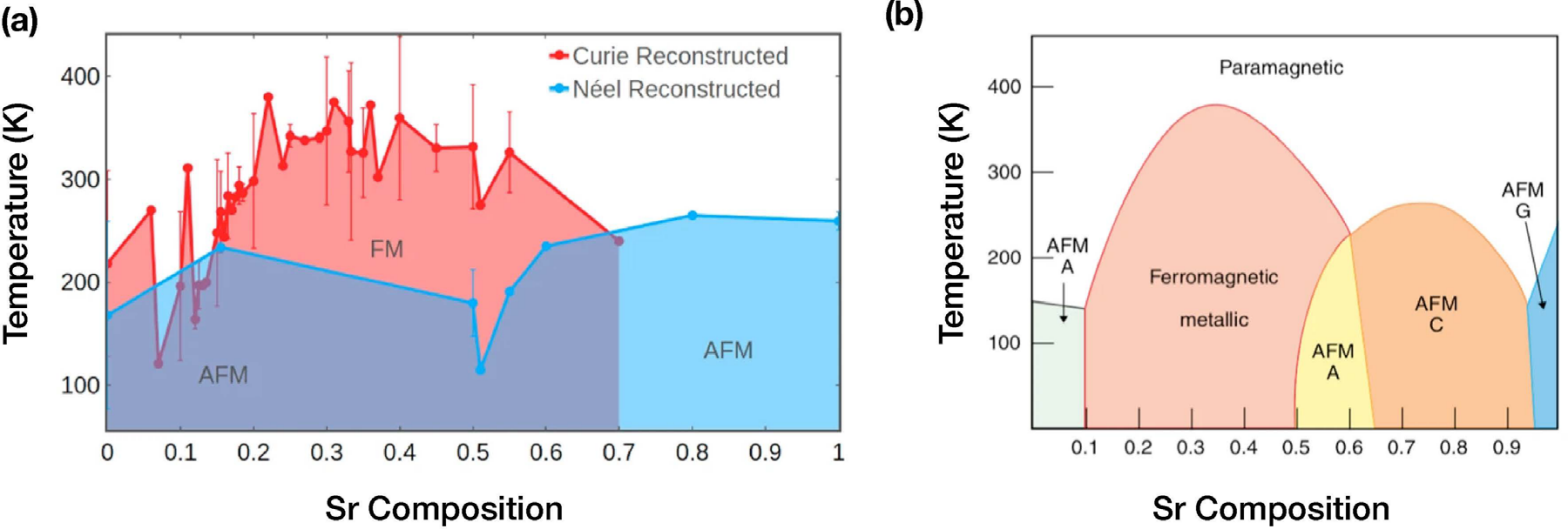
(a) point‐by‐point mapping of Curie and Néel temperatures as a function of strontium composition, x, in the family of La_1‐x_Sr_x_MnO_3_ magnetic materials using data mined from the academic literature [78]. (b) mapping of the magnetic phases of same chemical family created manually by an expert in the field [79]. Adapted from Court & Cole [78].

Figure 7b displays another phase‐space mapping of this same class of materials, which was manually crafted for a review article [79] using a wealth of domain expertise that was acquired from myriad years of experimental research on this class of magnetic materials. This map is naturally more comprehensive given the long‐standing domain expertise involved in its construct and the review type of publication from which it originates. Accordingly, Figure 7b can be regarded as the “model answer” in our understanding of the magnetic phases of La_1‐x_Sr_x_MnO_3_ materials, correct at the point of publication of the review.

The key point here, though, is that our data‐driven approach to this phase‐space mapping exercise reveals most of the magnetic domains shown in this review article, without recourse to many years of domain expertise, and utilizing data‐extraction methods that required just a few years of effort to produce the entire magnetic database**;** not just the data for this class of magnetic materials but for all types of magnetic materials. Accordingly, our database can be used to map the phase space of any known family of magnetic materials. This will perhaps be most beneficial for cases where no ‘model answer’ has yet been published as a manually collated phase‐space mapping.

However, this comes with the caveat that such mapping predictions would be reliable. Here, a comparative analysis of the two maps in Figure 7 provides a useful benchmark. The ‘model answer’ displays six magnetic phases for La_1‐x_Sr_x_MnO_3_ materials: one ferromagnetic, one paramagnetic and four antiferromagnetically distinct structural phases. Our data‐driven approach identifies only four distinct phases: the ferromagnetic phase, the paramagnetic phase, and two antiferromagnetic phases that cover the overall areas of the four antiferromagnetic phases that have been manually resolved, as demonstrated in Figure 7b. While our data‐driven approach does not resolve the ‘AFM A’ phase, the ChemDataExtractor‐generated result does suggest the presence of a distinct antiferromagnetic phase in this region of phase space, as shown by the substantial dip in the T_N_ profile that effectively bisects the antiferromagnetic phases at the composition, *x* ≈ 0.5.

If the ‘model answer’ shown in Figure 7b had not been known at the point by which the results of this data‐analytical study had been obtained, this dip would inform experimental researchers in magnetism that this compositional and temperature range would be a good region of phase space to explore to try to discover a new magnetic phase for this family of magnetic materials.

It is also important to note that the error bars shown in Figure 7a represent the standard deviation between *T*_C_ or *T*_N_ values of individually reported measurements that were mined for a given composition, x, where such data have been reported multiple times across different papers. This equips this data‐driven approach with a good level of uncertainty quantification. Furthermore, these errors directly take advantage of the sum‐that‐is‐greater‐than‐its‐parts aspects of manifold independent reporting of *T*_C_ or *T*_N_ values, which can occur in the field of magnetism. Indeed, the focus on such a type of error is prioritized over errors that stem from a single report, wheresoever multiple reporting of *T*_C_ or *T*_N_ values has been extracted by ChemDataExtractor. Naturally, the ChemDataExtractor‐generated magnetic database will not capture everything here, given that it did not mine every paper in existence across all publishers. Nonetheless, the comparative analysis of results from Figure 7a,b demonstrates that they can provide representative phase space mappings of magnetic materials.

While the mapping of phase space is the natural focus of this section of work, it is worth mentioning in its conclusion that regular line plots can also prove to be useful in the prediction of new phases of materials. Once again, we have taken an example from the field of magnetism, where a plot of lanthanide (Ln) radii versus ChemDataExtractor‐extracted Néel temperatures for LnMnO_3_ materials is shown in Figure 8 [78]. One can immediately observe the non‐linear dependence of *T*_N_ versus Ln radii within a new plot feature that evolves as materials contain heavier Ln ions beyond Ln = Dy or Tb. Indeed, this nonlinear trend is the manifestation of a structural transition, as was corroborated via an ML‐based regression analysis of these data that produced the blue‐colored reconstruction of the *T*_N_ profiles as a function of Ln radii in Figure 8 [78].

**FIGURE 8:**
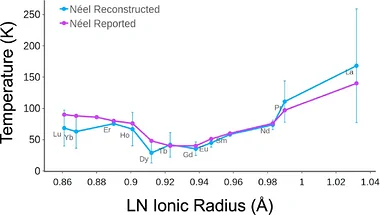
Nonlinear dependence of *T*_N_ versus rare‐earth ionic radii observed in LnMnO_3_ materials for rare‐earths heavier than gadolinium. Adapted from Court & Cole [78].

### 3.5 Data‐Enabled Forecasting and Decision‐Making for Materials Science

Another advantage of ‘chemistry‐aware’ auto‐generated database software tools, such as ChemDataExtractor, is that they can forecast future trends in materials science using the wealth of accumulated historical data as a basis. This is possible owing to the standard practice of tools, such as ChemDataExtractor, to tag the DOI of the paper from which each data record is sourced from an auto‐generated database. The motivation for establishing this standard practice arose from a wish to ensure good quality control of NLP‐generated databases, in that a user can then always have recourse to refer back to the original source document to manually validate any data entry within any ChemDataExtractor‐generated database, that is, to afford data traceability. However, the tagging of a DOI to each document offers a very useful latent benefit in that it contains the year of publication of each data record. Accordingly, users of these materials databases can plot materials‐science data as a function of the year in which they were reported. As such, data‐enabled forecasting of materials‐science trends is innately possible with ChemDataExtractor.

These forecasting capabilities are best illustrated via a case study, which is now discussed, using a ChemDataExtractor‐generated database about thermoelectric materials and their salient properties [55]. Figure 9 summarizes the contents of this thermoelectric materials database in the form of data distributions. Six histograms are presented, one for each thermoelectric property within the database: the figure‐of‐merit for the performance of a thermoelectric material, ZT; the Seebeck coefficient, S; electrical conductivity, *σ*; thermal conductivity, *κ*; temperature, T; and the power factor, PF.

**FIGURE 9:**
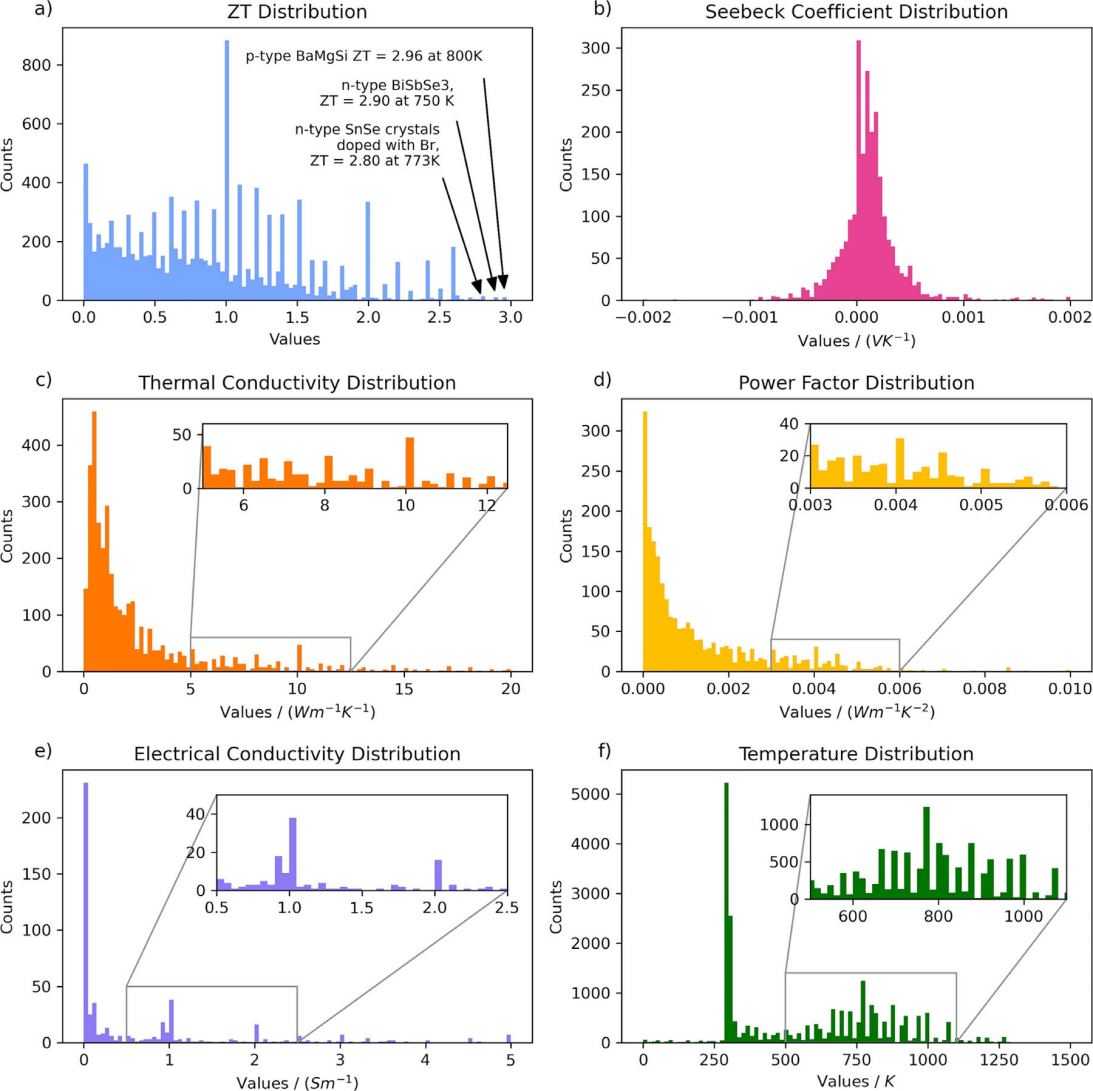
Statistical distributions of properties in a thermoelectric materials database created by ChemDataExtractor. Adapted from Sierepeklis & Cole [55].

The first five of these properties are related via the equation:

(6) $$\mathrm{ZT}=\frac{S^{2}\sigma T}{k}$$

Thereby, the higher the value of ZT, the higher the performance of its thermoelectric material.

Having factored in some practical constraints concerning the spikes observed in these data that arise from experimental rounding error (see Box 2), the intrinsic forecasting ability of the data within these histograms can be evaluated. Figure 9 shows that ZT is the most frequently extracted thermoelectric property. This stands to reason, given that ZT is perhaps the most useful metric to report, as it directly and simply informs a researcher about the performance of a thermoelectric material. Indeed, the annotation on this histogram of ZT in Figure 9 shows that one can simply use it to read off information that identifies, say, the thermoelectric materials that have some of the highest reported performance characteristics to date. One could of course argue that a domain expert in thermoelectric materials might already know this, while a non‐expert could perhaps use an internet search tool, such as ChatGPT [1], to find this information. However, a key utility of this thermoelectric materials database is that it can be used to perform explicit technological forecasting, as is now exemplified.

Figure 10 shows two forms of ZT characteristics for thermoelectric materials plotted as a function of the year that they were reported. The use of descriptive statistics to showcase these historical trends in ZT enables a technological forecasting of the industrial prospects of thermoelectric materials. This forecasting capability of the database provides a data‐driven decision‐making vehicle for strategists who are involved in horizon scanning of the thermoelectric research landscape.

**FIGURE 10:**
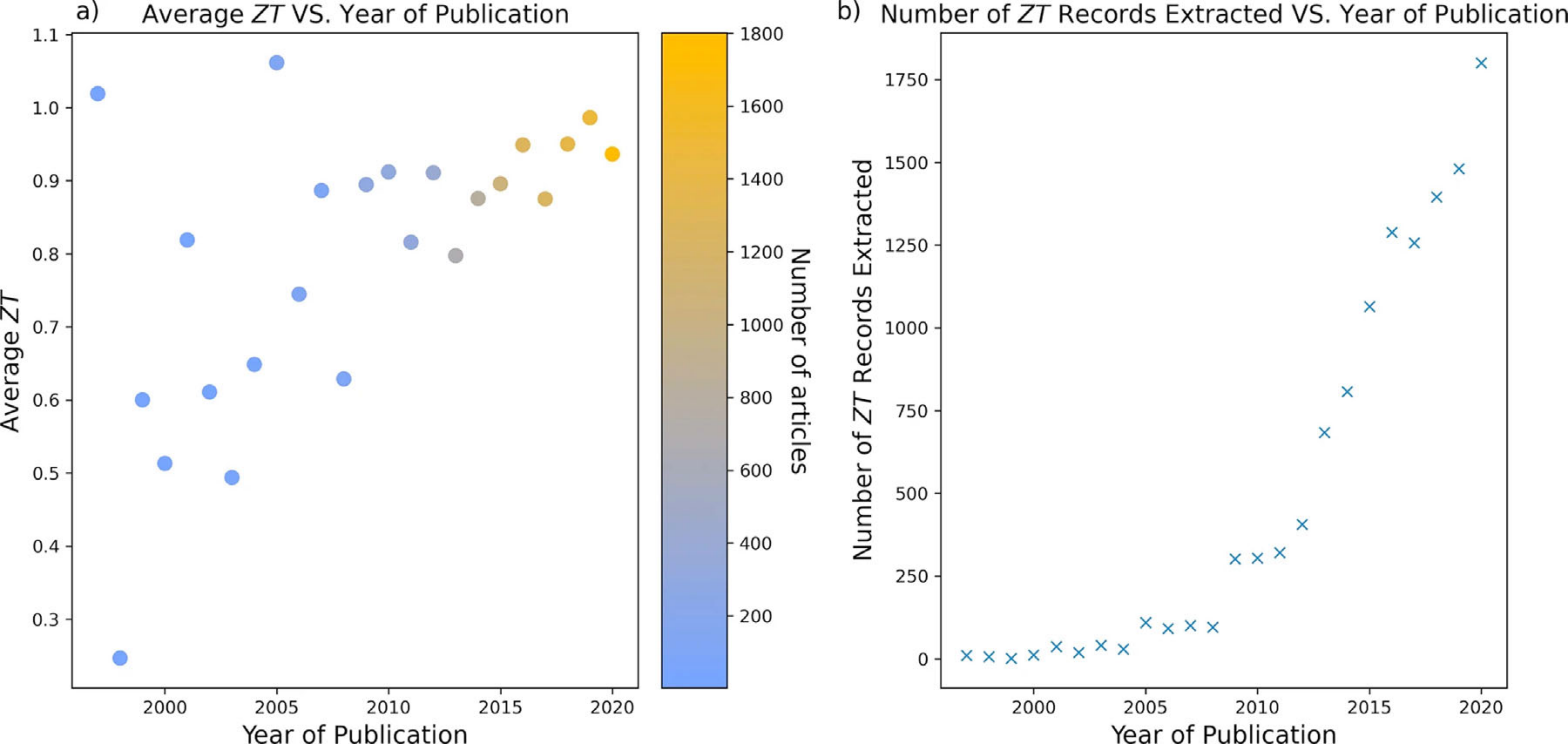
Forecasting trends of the (left) average ZT or (right) number of ZT records of a material extracted from the academic literature as a function of the year of publication. Adapted from Sierepeklis & Cole [55].

For example, Figure 10a shows how the average ZT value of thermoelectric materials has essentially increased linearly over the years; as such, one can extrapolate using a linear fit to the plot data to predict that the average ZT of thermoelectric materials will reach a value of 1.5 by the year 2052. Meanwhile, Figure 10b reveals how the number of ZT values that are being reported in publications has increased over the years. The sharp and highly non‐linear growth observed in this reporting of ZT values naturally reflects a rapidly increasing interest in thermoelectric materials research, which is perhaps not so surprising given that it represents an energy‐sustainable technology. However, these data allow the industrial strategist to *quantify* the likely level of future interest in thermoelectric materials. This can be very useful in the decision‐making process for an organization that is wondering if they should invest in thermoelectric materials research and to what extent they should invest. Such decision‐making opportunities are naturally not unique to this case study. Indeed, one might even use multiple ChemDataExtractor‐generated databases that span a wider range of energy‐sustainable technologies, to forecast the best strategic energy mix of research and development (R&D) investments for a given corporation in going forward over the next few years. Data‐driven decision‐making approaches that can inform organizations about R&D strategies which stand to create the best energy mix for affording energy sustainability are extremely relevant, given the urgent global need to realize Net Zero as soon as possible.

**Box 2. Systematic rounding error observed in experimental materials databases mined from the scientific literature**

Note that all data distributions shown in Figure 9 display spikes which pertain to a practical point of interest. These spikes occur over a sequence of unitary incrementing values for a given significant figure. For example, the main spikes in the histogram for ZT lie at 1.0, 1.1, 1.2, ….; while the highest peak in the inset of the histogram for power factor lies at 0.003, 0.0035, 0.004, and 00045; the main spikes in the histogram for *σ* lie at 0, 1, and 2.

These spikes represent the simple consequence that experimental data exhibit *rounding error*. This is because experimental data about properties are only ever reported up to a certain number of significant figures, and this number tends to be the same for a given property, perhaps due to the level of accuracy by which they can be measured on standard metrological instrumentation, or are simply an accepted in reporting conventions.

Given the large amount of data present in each of the six histograms, one could assume that the central limit theorem holds and, as such, could be used to smooth out these spikes by placing Gaussian distributions over them. However, we prefer to report ChemDataExtractor‐generated data faithfully so that a user receives the unadulterated information; that way, users of our databases have the maximum versatility in how they apply a database for their bespoke purposes; for example, they could choose to apply the aforementioned data smoothing option for themselves, or perform any other manipulation strategies as per their wishes.

### 3.6 AI‐Interactive Querying of Data About Materials for Energy Science

Chemistry‐aware data‐extraction tools, such as ChemDataExtractor, have proven to be effective in auto‐generating custom materials databases that contain domain‐specific chemical information. However, there are increasingly more demands on the research community for more complex types of information; such information may exist in the literature, or it may not, and yet it could be surmised from being exposed to a large quantity of sufficiently relevant literature. To this end, the context of sentences becomes an important commodity, as does the means by which one can obtain such semantic knowledge. Language models are naturally predisposed to learning such contextual knowledge and applying it, once suitably fine‐tuned, to serve a given downstream task. The amenability of LLMs to deliver on such tasks to solve materials‐science challenges is now illustrated through a case study on battery research.

In this case study, ChemDataExtractor had been used to auto‐generate a materials database about battery devices. Figure 11 displays the contents of this database in the form of data distributions of the five main device characteristics that are typically reported for battery materials: voltage, capacity, conductivity, energy, and Coulombic efficiency [53]. Incidentally, these data distributions offer another illustration of the aforementioned effects of rounding error via the observed spikes, especially at the integral or half‐integral values of the voltage or the exponent of conductivity (*cf*. Box 2).

**FIGURE 11:**
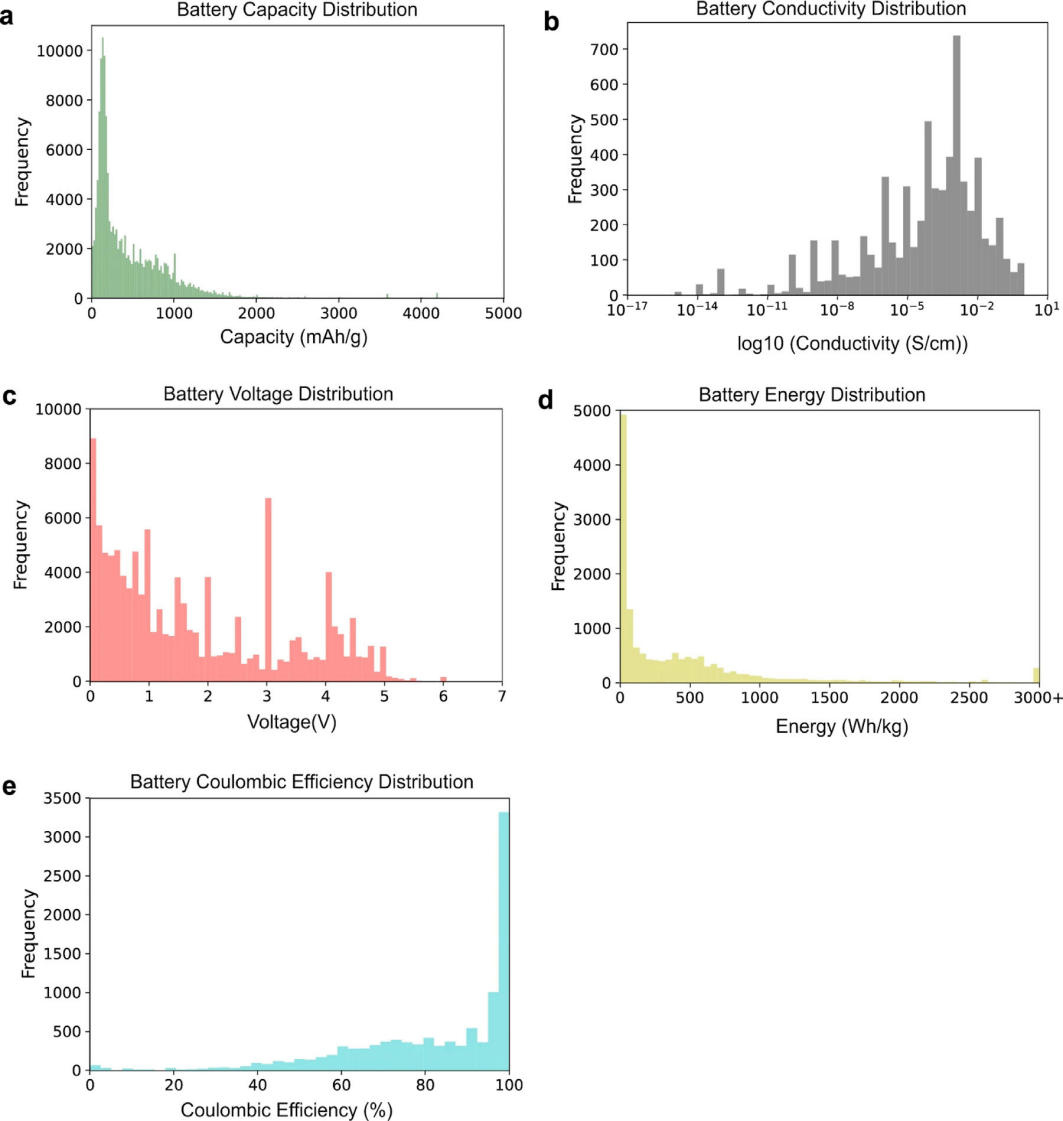
Statistical distributions of device characteristics in a battery materials database created by ChemDataExtractor: (a) capacity; (b) conductivity; (c) voltage; (d) energy; (e) Coulombic efficiency. Adapted from Huang & Cole [53].

One key challenge surfaced during the course of this work: while ChemDataExtractor was able to tag chemical names to their device characteristics, it had no means to determine whether a given chemical corresponded to an anode, cathode or electrolyte function of its associated battery. Fortunately, we were able to solve this materials‐classification challenge by training and applying LLMs via the following steps (*cf*. Section 2.2 for details on training LLMs).

First, we created a domain‐specific LLM for battery materials, which became known as BatteryBERT [37]. This LLM resulted from pre‐training a vanilla BERT‐base LLM on an input corpus about battery material devices. This corpus included all papers that had contributed to the data records in the aforementioned ChemDataExtractor‐generated database on battery material devices. This afforded a battery‐rich corpus to help gear the LLM to learn domain‐specific capabilities in battery research.

Next, a span‐classification layer was added on top of the fine‐tuning stage to search text for keywords: anode, cathode, and electrolyte. This provided context for the subsequent step in the operational architecture of the BatteryBERT language model, where it was fine‐tuned for downstream question‐answering (QA) tasks that could determine whether a battery material pertained to an anode, cathode, or electrolyte function. This fine‐tuning process was made possible by training one extra fully‐connected neural network layer of the BERT model using the combination of two datasets that comprise contextual pairs of questions and answers. One of these QA datasets is the well‐established Stanford Question Answering Database (SQuAD) version 1.1, which contains > 100 000 QA pairs that have been constructed from generic English‐language text [80]. We manually curated the other QA dataset, which was tailored specifically to our aforementioned materials‐classification challenge. Thereby, key questions were: *“What is the anode?”*, *“What is the cathode?”*, *“What is the electrolyte?”* While this QA dataset was small, containing only 272 data records, its high level of specificity brought significant impact to the fine‐tuning exercise.

Having fine‐tuned our BatteryBERT model for this battery‐specific QA task, the three aforementioned questions were asked of the relevant text in papers from the battery‐rich corpus. The answers determined if the chemical extracted from this text in each paper pertained to an anode, cathode or electrolyte. These answers were fed back into the original ChemDataExtractor‐generated battery device database as a new entry for each chemical record, thereby augmenting the database with a classification of each chemical as being part of an anode, cathode or electrolyte. This QA‐based LLM‐classification approach was deemed to be highly successful, judging from how well the eight most frequently reported anode, cathode and electrolyte materials for batteries were classified according to expectations (see Figure 12). Thus, we solved our materials‐classification challenge.

**FIGURE 12:**
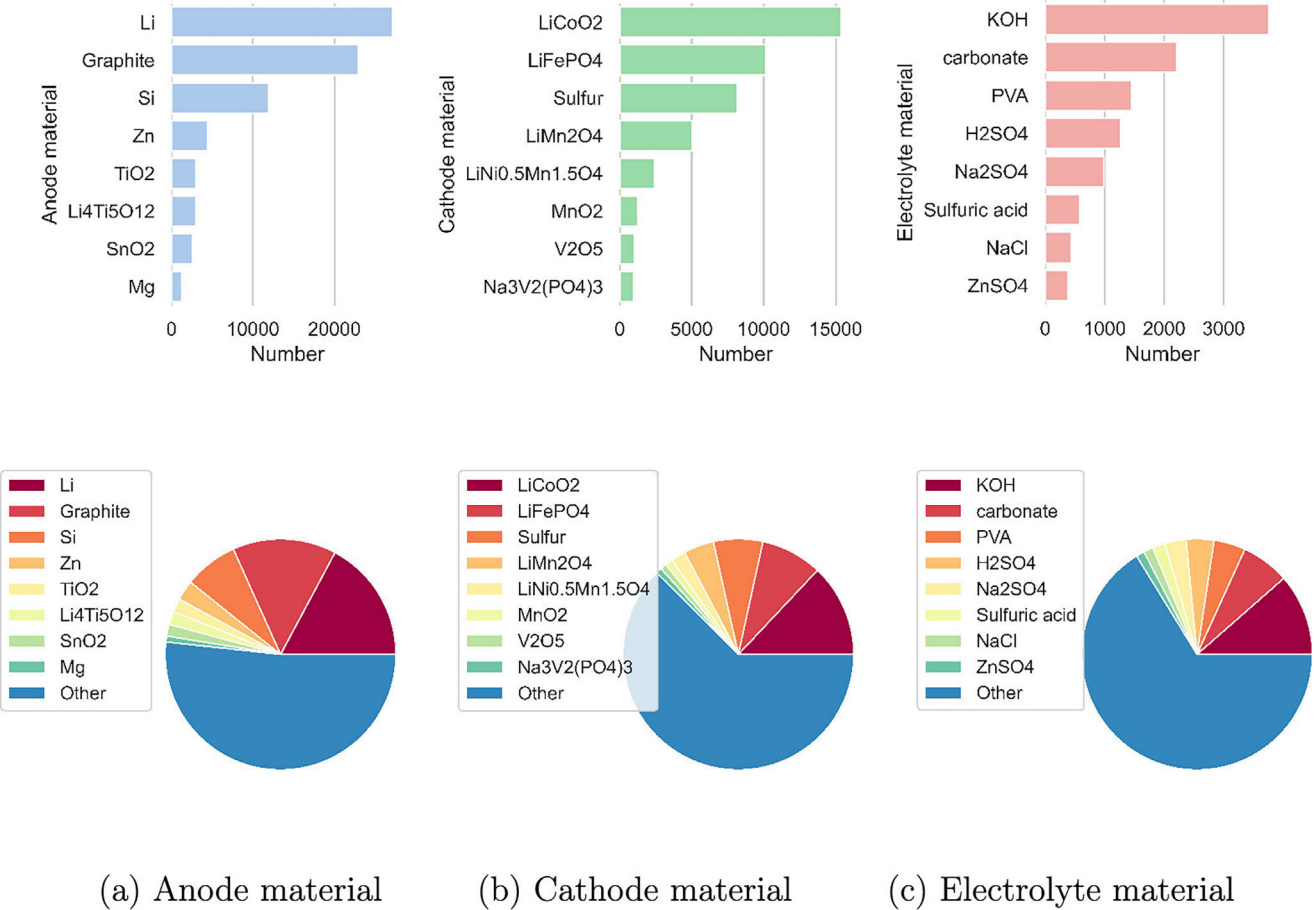
Classification of battery materials from a ChemDataExtractor‐generated database as an (a) anode, (b) cathode, or (c) electrolyte material using the materials‐domain‐specific BatteryBERT language model that had been pretrained and fine‐tuned using SQuAD and battery‐specific pairs of questions and answers. Adapted from Huang & Cole [37].

This case study refers to the first published report of a BERT model that was pre‐trained for a specific field of materials science. Thus, we manually curated a small dataset of 272 QA pairs for this task. Our methodologies for fine‐tuning domain‐specific LLMs have since advanced considerably, such that we can now auto‐generate much larger datasets of QA pairs from materials datasets that have been produced using ChemDataExtractor. This was achieved by developing and deploying an extension of knowledge distillation methods, which are discussed in section 4.2.1. A key practical advantage of auto‐generating large datasets of QA pairs from conventional materials datasets is the ability of the former to be deployed in automated high‐throughput prompt engineering tasks. Such tasks can help to solve domain‐specific materials‐centered problems using the latest advances in agentic AI (see Section 4.3).

## 4 The Future

The AI revolution is moving so fast that it is hard to keep abreast of the present innovations, let alone consider the future. Nonetheless, there are clear patterns in the evolution of new developments in AI for materials science from which future opportunities can be observed. These opportunities will be considered in a bipartite manner: the evolving framework that AI uses to interact with knowledge, and the evolving challenges and opportunities surrounding the data that AI needs to become ‘intelligent’.

The framework that AI uses to interact with knowledge is captured by Figure 13. Thereby, the world is witnessing a progressive shift from analyzing data using static datasets, to a more interactive querying of data that are held in language models, to analyzing multiple types of data concurrently using multi‐modal language models, and most recently using more explicit query‐based data input to fuel decision‐making workflows. This section discusses this evolution by first comparing the relative advantages and disadvantages of using static datasets versus large language models. The rise of small language models is then considered to two ends: to help democratize AI for materials science, and to offset climate change by circumventing the currently exorbitant costs of training domain‐specific language models. The use of small language models as AI agents to automate materials processes and provide highly interactive human‐in‐the‐loop AI is then discussed. The challenges and opportunities brought about by using the combined strength of multiple types of materials‐data streams within multi‐modal language models are then considered.

**FIGURE 13:**
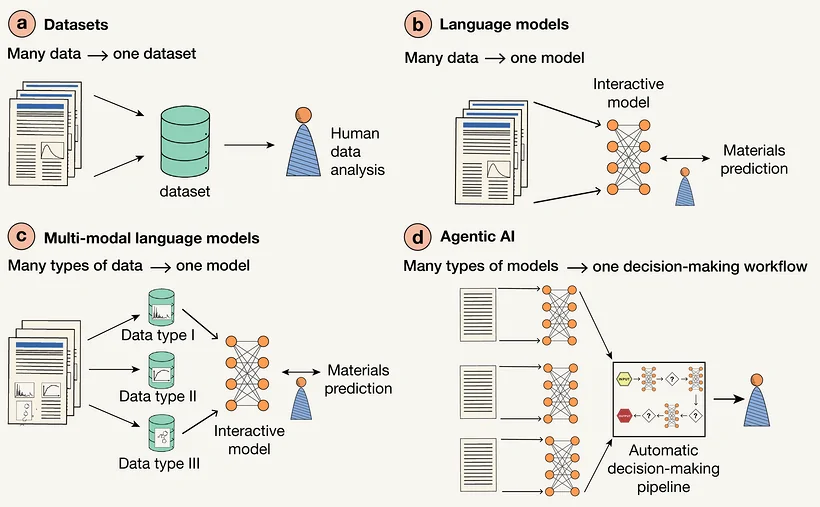
The progressive evolution of framing knowledge for successions of AI paradigms: from (a) materials databases, to (b) language models, to (c) multi‐modal language models, to (d) AI agent‐driven decision‐making workflows.

This section then discusses the data that AI needs to become ‘intelligent’ in materials science: in terms of legacy data that can be categorized into various forms of ‘dark data’; and how we can move forward with new approaches that overcome these data challenges in AI for materials science in the energy sector. Thereby, we have a golden opportunity to fully harness the opportunity of AI for materials science by delivering the best materials data for the future.

### 4.1 Literature Mining With NLP or LLMs?

QA‐based LLM approaches have also been used to extract materials data from the literature, without the aid of ‘chemistry‐aware’ NLP methods, using much larger LLMs, such as GPTs; as was noted in Section 2.2. Such approaches typically employ various forms of prompt‐engineering. These represent exciting developments in materials science, although some of these GPT‐based prompt‐engineering approaches suffer from low‐quality data extraction owing to GPT's lack of a domain‐specific focus [81]. Higher quality can be achieved if greater sophistication is built into the prompt‐engineering system, despite GPT's lack of domain‐specific focus [82]. Indeed, Section 2.2 discussed some such efforts that have even been shown to produce materials databases with high precision [51], However it is important to bear in mind that such approaches tend to be geared to work where {material, value, unit} tuples exist within short‐sentence clusters of text [51], as such, they will tend to miss a lot of data that could be extracted from the literature, unless they are equipped with bespoke ‘chemistry‐aware’ capabilities.

This low recall issue contrasts with specialist tools, such as those that combine named‐entity recognition with relation extraction for fine‐tuning LLMs [49]. ChemDataExtractor version 2.1 and beyond also incorporates a BERT model for its chemical named‐entity recognition [21]. Furthermore, the Cole group has recently shown that concerted NLP/LLM approaches can also be used in sequence; thereby, ChemDataExtractor‐generated materials databases are now being reverse‐engineered to help generate large QA datasets for fine‐tuning LLMs, to aid prompt‐engineering applications in the energy sector [40, 41]. This emerging area of NLP/LLM hybrid data‐extraction solutions translates knowledge‐distillation methods [46] to materials science, as will be described further in the next sub‐section.

#### 4.1.1 Quality Control for Training LLMs

The required accuracy of an experimental materials dataset can often be a key deciding factor when selecting whether an NLP or LLM approach is the best data‐extraction option to solve a given problem at hand. Quality‐control considerations for the NLP‐generated extraction of experimental data were discussed in Section 3.1. Quality control of LLMs is also important.

The quality control of LLMs that have been trained for a particular materials‐science domain is much more difficult to assess, given that they afford probabilistic rather than deterministic output. Nonetheless, their efficacy can be checked indirectly by evaluating the strength of certain parts of the pipeline that were used to develop a given LLM. To this end, the following considerations offer some basic guidance:

- The demographics of the corpus used to train an LLM can be classified; for example, the size of the corpus, the proportion of a subject area that is represented therein, and the level of overlap between adjacent subject areas that are represented therein.
- Loss convergence graphs associated with pre‐training LLMs demonstrate the extent to which an LLM is stable; albeit, this is not a measure of the *quality* of an LLM with regard to a target application.
- The layers in an attention head of a neural network can be visualized to gauge how well the context of text is learnt by an LLM; for instance, BertViz provides such a tool for the BERT family of LLMs [83].
- The effectiveness of fine‐tuning an LLM on downstream tasks can be evaluated using precision, recall, and F1‐score metrics to compare the results of the downstream task operations against their expected outcomes; for example, the expected reliability of an LLM on a QA downstream task can be tested against the known reference dataset of Q and A pairs [37, 38].
- The Expected Calibration Error (ECE) can be used to evaluate the extent to which a user can have confidence in an answer given by an LLM that has been fine‐tuned on QA downstream tasks. This ECE metric compares how well the estimated probabilities produced by an LLM match its observed probabilities on a given test set [84].

#### 4.1.2 LLMs as Data Resources Rather Than Materials Databases?

LLMs are increasingly being used as a core substitute for materials databases, especially in cases where the training of an LLM has been geared to a specific materials domain. It is difficult to advise on whether a materials database or materials‐domain‐specific LLM is a better information source to solve a given materials‐science problem. This is because the development of domain‐specific LLMs pertains to a very fast‐moving field, and because a given materials‐science task can be so different from another that decisions can only be made on a case‐by‐case basis. Nonetheless, this review introduces a basic guide to facilitate current decision‐making processes to this end, especially factoring in important practical needs of industry. This ‘3T formalism’ stands for “Timely, Tailorable, Tinkerable”, which is explained in Box 3.

**Box 3. How to decide if a materials database or materials‐domain‐specific LLM is best suited as the data‐extraction tool to solve a given problem in materials science**

There are, of course, areas of materials science that are not covered by these existing ‘big data’ resources. One then needs to decide if a new database or LLM needs to be specifically crafted to tackle the problem at hand, or if a general‐purpose LLM that has been pre‐trained on generic English‐language text, such as ChatGPT, can serve the given need. To this end, it is helpful to consider these needs according to a ‘3T’ formalism, “Timely, Tailorable, Tinkerable”, that this review introduces to facilitate the decision‐making process, especially factoring in key needs of industry. Thereby, one can ask the question: does the ‘big data’ resource need to be:

***Timely***. The time that it takes to create a large, high‐quality, materials‐science database by learning and applying a ‘chemistry‐aware’ text‐mining tool, such as ChemDataExtractor, will vary depending on the problem at hand and level of expertise of its user: a complex problem may take a novice 1‐2 years to realize a high‐quality dataset, while an expert may only need a few months to furnish the same result. Lower quality databases can be auto‐generated more rapidly if one does not invest in sufficient time to become an expert; although, an LLM may then be more appropriate if the quality of data is not so critical in solving a given problem.

***Tailorable***. A key factor in determining whether a ‘big data’ resource should be a materials database or an LLM concerns the level of detail in the data source that is needed to solve a given problem. For example, top‐level information is readily extractable using an LLM; to secure such data, there is little advantage in training an LLM for a domain‐specific task. By stark contrast, LLMs that have been trained on a domain‐specific materials‐science corpus ***excel*** when more subtle or complex types of information within a given domain are sought. For example, the top‐level keywords that describe an entire subject area, such as “photocatalysis,” can be extracted readily on a foundation LLM; yet, a deeper‐level keyword such as “co‐catalyst” requires an LLM that has been tailored to this subject area [43].

***Tinkerable***. The decision as to whether or not to use a database or an LLM naturally rests with the type of task at hand. The following sections will showcase examples of both types, although one simple decision‐making criterion is whether one needs to enquire upon a dynamic data landscape in an interactive fashion (which would favor the use of an LLM) or if an ML task simply requires static data as input (where a database would be more suitable).

### 4.2 Democratizing AI to Offset Climate Change: The Rise of Small Language Models

The global adoption of AI is fostering a seemingly inexorable rise in energy consumption, with myriad data centers being built around the world in efforts to cope with global demand. This rise in energy demand is not sustainable, a consideration that is even acknowledged by some of those developing AI methods. To this end, an increasing number of researchers are adopting small language models (SLMs), rather than LLMs, in their latest AI innovations. This shift in focus has been aided by recent findings that the performance of foundation LLMs can be readily outstripped by far smaller, highly targeted, domain‐specific language models [40, 41, 85, 86]. Pending such a SLM is small enough, it can function as a standalone entity, circumventing the dependency of LLMs to function via the cloud. This ensures privacy when using language models, easier automation when incorporating language models into off‐line pipelines, and encourages democratization of language models by disconnecting them from the cloud and stunting their energy demands.

These functional distinctions effectively define whether a language model is an SLM or LLM. Nonetheless, model size and computing demands are determining factors, and there is broad consensus that the language‐model characteristics shown in Table 1 distinguish SLMs and LLMs. A more detailed survey of SLM characteristics is given by Lu et al. [87].

**TABLE 1:** Distinguishing characteristics of small and large language models.^a^

Characteristic of language model (LM)	Small language model (SLM)	Large language model (LLM)
Number of model parameters	0.1–15 billion^b^	>30 billion
Computer runtime memory	Small (typically 0.2–12.5 Gb)^c^	Large (typically >50 Gb of VRAM)
Inference latency	Low (can be as low as 5 ms)	Moderate to high (a lot of effort is made to reduce this to < 200 ms to be useful in real‐time applications)
Training data (number of tokens)	< 150 000 tokens	Very large and continuing to rise (up to 1.5 × 10^14^ tokens, to date)^d^
Host for LM deployment	Edge devices (e.g., laptops, drones, robots, wearables, smartphones)	Cloud, data centers
Typical uses	Specialized, domain‐specific tasks; on‐device tasks, for example, phone apps Tasks needing fast response time, for example, real‐time text translation	Generic question‐answering tasks, broad, high‐level surveys
Well‐known examples	Microsoft Phi‐3‐mini & Phi‐3‐small, Google Gemma 3n, TinyLLaMA, Mistral 7B, BERT‐base, DistilBERT	OpenAI ChatGPT with GPT‐4 or 5 Google Gemini Ultra, Anthropic Claude Mythos 5, Opus, LLaMA3 (70B variant)

^a^ Based on typical current values. This SLM/LLM classification should be regarded as indicative rather than a universal threshold, given that the architecture, precision, context length, hardware, and training protocol of an LM affect its characteristics.

^b^ Some reports set the maximum to 5 or 10 billion, but Phi‐3 medium (parameter count: 14 billion) is formally classed as an SLM, so the ceiling is set here to include this model.

^c^ This range does not account for the use of compression methods such as quantization, pruning, or knowledge distillation (see [88] for a detailed survey on these effects on SLMs).

^d^ This upper limit at the time of writing concerns the gpt‐oss‐120b model.

These developments have key implications for three key areas of AI research: AI security and trust, AI agents and automation, and the democratization of AI. This third area of research is also being influenced by recent developments in knowledge‐distillation methods that are further reducing the energy demands of language models in a more substantial and fundamental way.

#### 4.2.1 Circumventing the Need to Pretrain Language Models via Knowledge‐Distillation Methods

By far, the largest energy demands of language models arise from when they are pretrained. Strubell et al. [44] calculated that the pretraining of a language model as small as the BERT‐base architecture will cost 1438 lbs of CO_2_ emissions, which is comparable to the CO_2_ emissions expended by a passenger flying from New York to San Francisco (1984 lbs). Yet, this estimate is a lower bound; it reflects a single training run and excludes the computational tests that precede it (the hyperparameter searches, failed runs, and ablations), which Strubell et al. note can multiply such costs considerably [44]. Moreover, BERT‐base language models (containing 110 M model parameters) are defined as one of the smallest SLMs nowadays, cf. Table 1, where model parameters of an SLM range from 0.1–15 billion. Compare this to a modestly scaled up mid‐sized SLM with 7B model parameters, whose pretraining requirements will cost 230 000 lbs of CO_2_ emissions (roughly 160 times more) [89]. So, the sustainability advantage of SLMs is comparative rather than absolute; the figures climb steeply with scale. Furthermore, a 32B variant of the same type of language model (a low‐end LLM according to Table 1) will emit roughly 1.1 million lbs of CO_2_ from the pretraining step alone [89], and as the field shifts toward ‘reasoning’ models, the cost climbs further. At the post‐training or fine‐tuning stage, this 32B reasoning variant will consume 17 times more data‐center energy than its instruction‐tuned counterpart, owing to the reinforcement‐learning iterative “thinking” process [89]. These cited examples are naturally case‐specific estimates of this sustainability issue and they do not represent a universal consequence of model size since manifold LM characteristics can affect the amount of CO_2_ emissions. Moreover, while the sustainability footprint of creating an LLM is particularly affected by its pretraining stage, the entire pretraining model‐development process, as well as post‐training (e.g., fine‐tuning) processes, all contribute to the overall CO_2_ emissions overhead of an LLM. Nonetheless, these case studies illustrate the problem. It is therefore not surprising that so many data centers are being built to accommodate the AI revolution. However, the development of energy‐efficient AI methods is starting to challenge this need.

Knowledge‐distillation methods [46] are proving particularly useful in this regard. Within the field of materials science, Cole et al. have developed a knowledge‐distillation method that is symbiotic to ChemDataExtractor‐generated materials databases [40, 41, 85]. This extends the conventional model‐to‐model mapping paradigm in knowledge distillation to a data‐to‐model mapping. Thereby, the ‘power of knowledge’ is placed outside a language model, whose fine‐tuned process is exploited as a data‐to‐model interface. The workflow of our method is shown in Figure 14a; in essence, it auto‐generates myriad materials‐domain‐specific QA prompts by ‘reverse engineering’ ChemDataExtractor‐generated materials databases. This involves reframing the original context from which each data record was extracted as material‐centric and property‐centric questions; and pairing these questions with answers that comprise the material and property information within each data record. This affords a materials‐domain‐specific QA dataset that typically consists of several hundred thousand QA pairs. These domain‐specific QA pairs can then be used to fine‐tune a vanilla language model for that materials domain.

**FIGURE 14:**
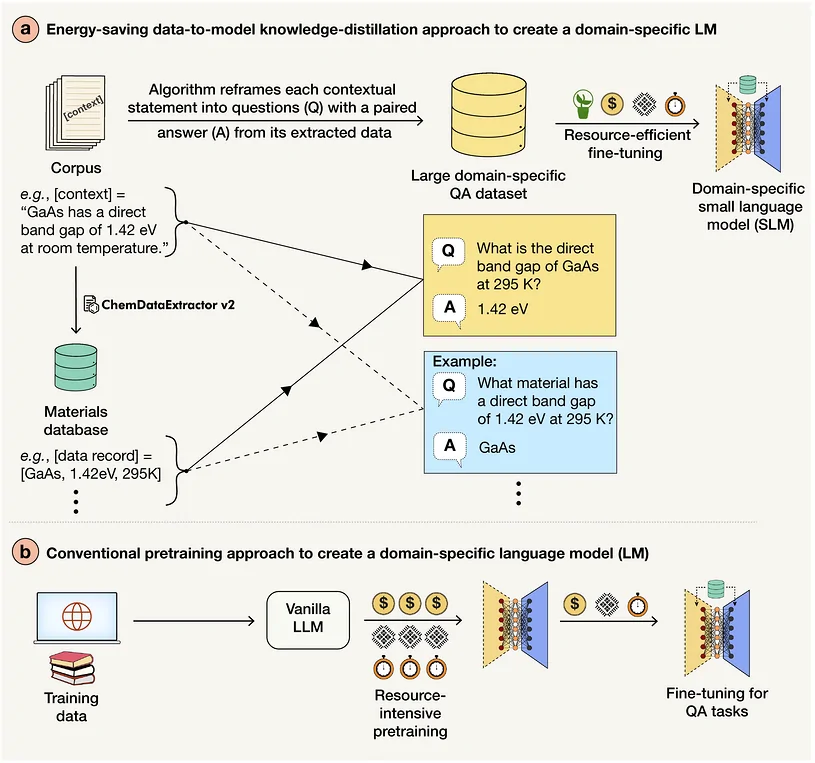
(a) An energy‐saving knowledge‐distillation approach to creating domain‐specific language models, by ‘reverse engineering’ ChemDataExtractor‐generated materials databases. (b) The conventional approach to pretrain a domain‐specific language model, which is far more resource‐intensive in terms of financial cost, computing demands, and training time.

The resulting performance of the language model is marginally less than that of the same vanilla language model which has first been fully pretrained using an entire domain‐specific corpus (Figure 14b); yet, the former is realized without the massive energy demands of a full language‐model pretraining process. Therefore, our method provides a way to develop language models without the need for vast energy overheads, such as supercomputers and data centers, which are inaccessible to most of the global population; i.e., this method can democratize the development of domain‐specific language models for custom applications.

Given that domain‐specific language models are built from rich, condensed information sources, they tend to be smaller in size than general‐purpose language models; such that they are commonly classified as an SLM. This smaller size makes domain‐specific language models highly versatile. For example, it was mentioned earlier (Section 2.2) that SLMs could be particularly useful in the development and deployment of AI agents for autonomous operations; a topical case in materials science being the embedding of AI agents with digital assistants to deliver specific goals, as will now be discussed.

### 4.3 AI Agents and Automation

AI agents are digital assistants, most of which have been designed to help a human deliver a range of goals automatically. Current AI agents tend to function with a core LLM that has an ability to call various tools to help deliver its goals provided by the human (the ‘user’).

State‐of‐the‐art digital assistants for materials‐science applications tend to rely on an AI agent that is driven by a GPT‐based LLM, but which engages in a set of ancillary computational tools to deliver specific goals. The scientific goals of such AI agents currently include:

#### 4.3.1 Chemical Synthesis and Safety Planning

This type of AI agent aims to help the human design and execute chemical reactions in a more optimized fashion and in a semi‐automated fashion. The AI agent may offer the human user a variety of advice, ranging from recommending reaction conditions to them, predicting a synthetic product, to informing them about the chemical safety of a given synthetic plan. Three prominent examples of this type of AI agent are ChemCrow [90], Coscientist [91], and ASKCOS [92].

#### 4.3.2 Agentic Self‐Driving Laboratories (SDLs)

This type of AI agent is able to autonomously run an entire experiment within a laboratory via a closed‐loop process, that is, it proposes how to run an experiment based on a materials‐science objective that is set by the human; it then executes one or more series of experiments, using the results from each experimental run to update its procedure to run more optimally until it delivers the result according to the set objective.

A comprehensive review on SDLs for materials science and chemical applications is given by Tom et al. [93], to which the reader is referred for full details. They classify levels of automation in the same way that the extent of automation in self‐driving cars is categorized [94]. This alignment between two self‐driving technologies is helpful, as self‐driving cars offer a publicly familiar gauge from which SDL levels can be readily understood. Thereby, an SDL with level 5 capabilities corresponds to a fully automated SDL with generative AI software being fully integrated into entirely automated SDL hardware. In practice, Level 4 SDLs that are automated with ‘human‐in‐the‐loop’ tend to be preferable [95]. Indeed, level 5 SDLs are deemed to be currently unattainable [93] while successful level 4 SDLs have already been realized for a few robotic experimental set ups; examples include the photocatalyzed hydrogen production from water [96], the AI‐driven materials discovery of a new metal‐organic framework that exhibits metallic conductivity [97], the data‐driven robotic synthesis of colloidal nanocrystals using a data‐mining process for its synthetic design [98], an automated materials characterization workflow using powder x‐ray diffraction [99] the autonomous running of laboratory experiments for quantum computing with closed‐loop feedback control [100], and the similarly automated running of an atomic force microscopy (AFM) experiment [101]. The authors of this AFM study showed results with varying levels of success, noting the need for benchmarking and safety protocols to be placed into the AI agentic workflow. Wang et al. also highlighted the importance of ensuring model reliability in these sorts of AI and data‐driven workflows, using a range of performance evaluation, domain of applicability, and uncertainty quantification methods [102]. Notwithstanding such practical challenges, these pioneering advances demonstrate the great potential that SDLs hold for energy‐sustainable materials science and chemical applications, which should be achieved pending considerably more development [101].

These SDL examples operate within one laboratory, although many experimental research challenges are not solved by working within such a level of confinement. Instead, experiments tend to evolve organically to meet a growing underpinning scientific need. Specialized research equipment and associated scientific expertise that can be literally spread across the world are often needed to solve a key research problem. Thereby, the development of distributed Agentic SDLs has been proposed to reflect this wider scope, so that the field of SDLs can start to realize its far greater potential [103].

One might suppose that the extent to which SDL operational workflows may be distributed or concentrated will ultimately adapt to experimental demands, as they become more versatile. Meanwhile, the bespoke tools that a given Agentic SDL calls to perform individual material functions as part of the ensemble SDL objective are likely to be more confined; as such, they can be developed in a modular fashion with a simple input/output (I/O) operation to interface with an overarching SDL agent. These automated tools are also currently under development.

#### 4.3.3 Automated Tools for Materials‐Science AI Agents

A bespoke set of tools that perform an automated function *at each part* of a full experimental supply chain is needed to operate an agentic SDL. For example, an agentic SDL that is set up as a ‘design‐to‐device’ pipeline for data‐driven materials discovery would need to call a range of modular tools with which it could interface in a simple fashion (e.g., I/O mode) to step through its supply chain in the typical order: materials design, property prediction, materials synthesis, materials characterization, materials manufacturing, materials device fabrication and testing. This agentic SDL would contain the experiment in a closed‐loop, with feedback control, to update and adapt the experimental procedure to optimize its objective of data‐driven materials discovery.

These modular tools may source third‐party data, perform internal calculations or simulations, or execute machine‐learning models or alike, so that they can deliver a response to the call by the SDL agent. In the example given, the SDL agent may first engage with external materials‐design software, relaying its output to a machine‐learning model that executes property predictions, which are fed to a robot that performs synthetic chemistry to produce a material that has been designed to exhibit the predicted property, which another tool characterizes. Pending the synthesized material meeting its designed specifications, the SDL agent will subsequently engage additional tools that will help it be manufactured for a device in which it will be incorporated and tested. If the result does not discover a material that has the user‐required specifications, a feedback loop within the agentic SDL conveys the experimental findings back to an earlier part of the ‘design‐to‐device’ supply chain; whereupon, the agentic SDL updates its experimental procedure in a way that learns from its errors, so that it can adapt itself for a subsequent run which aims to produce a more optimized material.

Tools that function automatically with a simple I/O operation for data‐driven materials discovery would be most efficient for agentic SDL operations. More slimline tools of this nature are needed that span the full ‘design‐to‐device’ pipeline, although automated materials‐property‐prediction tools and AI‐based materials‐characterization tools with simple I/O operations are starting to become available [104, 105, 106].

### 4.4 Concerted Modeling of Different Types of Data: The Rise of Multi‐Modal Models

While agentic SDLs access different types of data, they do so sequentially via a network of operations; as such, they do not implicitly account for correlated data, which can be crucial in the interpretation of materials‐science data. In contrast, multi‐modal models are implicitly designed to process different types of data within a concerted fashion so that correlated data are modelled explicitly.

Multi‐modal learning models (MMLMs) make this possible by feeding different types of data into different encoders, each of which processes a distinct representation of data (*e.g*., text, images, graphs, audio). In turn, these data feed a deep‐learning neural network to train a model so that its weights implicitly incorporate the correlated nature of the input data. Examples of MMLMs in materials science include: MatMCL that predicts mechanical properties of materials in a way that incorporates their correlated structure‐property‐processing relationships; [107] MultiMat that predicts crystal structures and their associated bulk modulus, elastic constants, and band gaps from diverse types of data that represent crystal structures, density‐of‐states, charge density, and text [108].

Multimodal transformer models have also been developed which differ from MMLMs in that the different types of encoded data are ‘tokenized’ so that they can be processed through a multimodal transformer. Examples of such models in materials science include: Cephalo, which employs multi‐modal vision‐language models to enable bio‐inspired materials design and analysis [109], and the general‐purpose FM4M multi‐modal model architecture developed by IBM [110].

Multimodal modelling for materials science is very much an emerging field. Nonetheless, its model developments are being somewhat hindered by a lack of availability and alignment of data streams with different modalities. A change in data‐access policies may help to alleviate this practical bottleneck to progress; in particular, federated data‐access schemes that have shown success in other industries offer a possible solution [111].

### 4.5 Accessing Data Across Chemical Space

4.5.1

A global digital transformation in materials scienced is needed. The viability of multimodal modelling is constrained by a need to meet the difficult challenge of soliciting cognate data of different modalities (image, text, graph, etc.) in a concerted fashion. One might naively assume that this is not such an issue in materials science, given that materials tend to be experimentally studied using multiple metrologies; for example, materials tend to be characterized using multiple techniques. Yet, the reality is pole opposite. This is because cognate materials data (and metadata) do not tend to be collated or stored together within a common data‐management framework. For the most part, materials data are still being stored on different computers that are spread across disparate parts of a given organization, with access permissions that are set by the experimentalist. Additionally, many data are still being stored without the appropriate metadata that allows the data to be useful to anyone except for the scientist that collected the data.

On the good side, an increasing number of organizations have acknowledged the value of their data and are consequently making steps to change this situation for data that are acquired in going forward, by investing in a digital transformation; yet, this is a large and time‐consuming task. This digital transformation also presents data‐security issues in terms of setting who should have access to data, pending an evaluation of their perceived value. Regulations that are too prescriptive will limit the full potential of a dataset, while completely open data cedes any intellectual property of the data. Federated data‐access schemes offer a halfway house in that they set conditions to data access in a way that the data owner retains control over who accesses the data and for what purpose they have such access. Certain sectors of the banking and health industries have already demonstrated success in deploying such schemes, which materials science could follow [111].

#### 4.5.2 Sunken Treasures of ‘Dark Data’ From Experimental Research in Materials Science

A salient aspect of the data‐driven materials science presented in this perspective article has involved the solicitation of experimental data from scientific literature. However, there exists a massive bias in any materials dataset that is generated from such a source because a high proportion of experimental data are not published. These are known as *dark data* (Figure 15).

**FIGURE 15:**
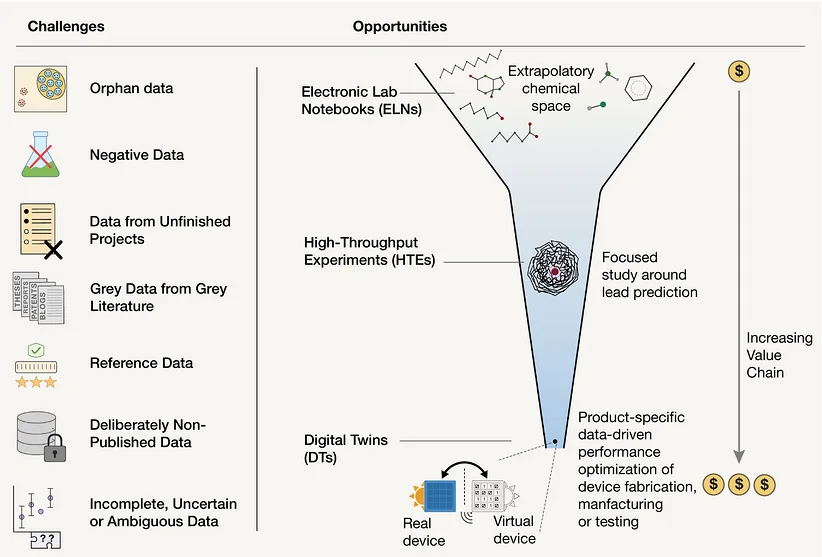
Challenges of accessing legacy experimental data about materials that constitute various types of dark data. Opportunities for accessing experimental data from laboratories in going forwards, via electronic laboratory notebooks, high‐throughput experiments, and digital twins.

Systemic and logistical issues have been cited as key reasons behind unpublished data, based on a report about United States National Science Foundation (NSF) data [112]. This same report estimated that full‐time doctorate researchers in NSF‐funded science and engineering disciplines at US academic institutions had accumulated unused data and chemical samples worth $6.2 billion in 2019. This total cost was apportioned into subject‐specific levels of unused resources: life sciences (63%), engineering (19%), physical sciences (10%), social sciences (8%). Furthermore, this total cost translated into an average of circa $29,000 per researcher based on NSF headcounts. This is concerning because the cost represents a significant contribution of the total US R&D budget for academic research, whose level of provision is increasingly under threat. Moreover, researchers around the world have tended to look to the US as an example to follow; thus, they will likely have mirrored this US behavior. This suggests that these levels of unpublished data may be reflected around the world.

‘Dark data’ therefore present a major issue for manifold reasons. Accordingly, they are classified into sub‐types according to the reason that they have not been included in a published scientific paper. These distinct types of ‘dark data’ are now described.

##### 4.5.2.1 Orphan Data

Researchers almost exclusively report experimental data in journal articles that pertain to successful scientific results, where success is defined by data that support the overarching hypothesis or prevailing argument of a given study. Many data from experiments are not published even though they have been acquired, processed correctly, and produce a positive scientific outcome, because they fail to deliver such support; these are known as ‘orphan’ data [112]. A common example of such data in materials science includes spectra from the materials characterization of an unintended chemical product. Their absence in a materials dataset imposes a systematic bias toward null scientific hypotheses that have been justified by data that are extant in scientific literature.

##### 4.5.2.2 Negative Data

Myriad experimental data are not published if they simply afford null results because an experiment could not fundamentally work. Common examples of such ‘negative’ data in materials science include null results from materials synthesis where it is empirically found that a chemical does not react with another under the experimental conditions explored.

There are currently few incentives for experimental scientists to publish negative data [112]. Yet, pending sets of negative experimental data have been acquired and processed correctly, they represent super‐important sources of data for constructing *balanced datasets* that comprise positive and negative data, in equal proportions ideally. The current dearth of balanced datasets about materials and their related properties means that many extant ML models that predict materials properties have been trained on datasets that carry a massive positive bias; a direct corollary is that many ML‐based property predictions carry systematic bias. Yet, the provision of negative experimental data could offset this bias by contributing to the production of balanced experimental datasets that are then used to train ML models for materials science that offer more informed results.

##### 4.5.2.3 Data From Unfinished Projects

Data that are eminently publishable but have been abandoned owing to the logistical issues that stem from unfinished work are, by far, the largest source of unpublished data; it is estimated that 82% of US academic scientists have hoarded data from unfinished projects [112]. The causal nature of such ‘dark data’ is somewhat different to the aforementioned forms of abandoned data, that is, orphan or negative (null) data, in that data from unfinished projects do not tend to be abandoned so deliberately. The act of abandoning data owing to logistical issues does not necessarily reflect an active choice to not publish; in contrast, one tends to make a choice to not publish orphan or negative data. This is a subtle, but important, point, because it suggests that data from unfinished projects could represent a hidden trove of positive scientific results which may uncover all sorts of scientific revelations.

##### 4.5.2.4 Grey Data from Grey Literature

Grey literature comprises documents that are published in a form that is not conventionally indexed by mainstream publishing houses or news channels [113]. Common examples of such work in materials science include scientific reports (*e.g*., annual reports, technical reports), blog posts, government documents (*e.g*., white papers, policy papers), non‐government public‐sector reports (*e.g*., from charities, universities), company reports or catalogues, consultancy reports, working papers of research (*e.g*., papers on arXiv, chemRxiv or bioRxiv repositories), dissertations or theses by research students (*e.g*., PhD or Master's degree research write‐ups; at least the former are likely to be held by the university library of the graduating student).

These forms of grey literature are likely to hold a trove of scientific research ‘grey data’ [114]. In particular, the working papers of research are likely to be a high‐value source of positive scientific results, much of which will be very new and thus potentially very innovative. Meanwhile, PhD theses will collectively mirror many of the data in these working papers, data from conventionally published work, as well as offer a major source of ‘abandoned data’, for example, orphan data, negative results, or those from unfinished projects. This is because a PhD thesis presents an in‐depth scientific study that has more latitude for null or orphan results, which can be tempered with positive results. The latter may also become ‘data from unfinished projects’ as a PhD student is generally expected to draft papers on the publishable work from their thesis; they may not do so, especially as many PhD students leave academia immediately following their PhD defense, so unless the PhD project is continued by another researcher, the research may not ever be published.

##### 4.5.2.5 Reference Data

Many experiments involve the collection of data from ‘reference’ or ‘standard’ samples which help to calibrate, normalize, verify or process data on the subject sample. Reference measurements can be compared to those of a subject sample on an absolute or relative scale.

The former involves experiments that calibrate a sample measurement to a fixed value (an *absolute reference*). This fixed value will have been a priori determined by a dedicated quality‐control procedure that sets a standard scale for a given type of experimental method. A well‐known experimental methodology or instrument, whose operation depends upon an absolute reference value, tends to employ a certified reference material (CRM) [115]. This CRM has been externally verified to guarantee a standard (absolute) experimental value for the property being measured, according to a specified minimum accuracy and uncertainty that is traceable to a reference that defines a regulatory standard, for example, an International Organisation for Standardization (ISO) value. A common example of such ‘absolute reference data’ in materials science concerns the acquisition of x‐ray absorption spectra from a metal foil to calibrate the energy of an x‐ray absorption edge for a metallic element [115] that is a chemical constituent of the subject sample, whose local structural environment is to be probed. In the case of a highly specialized experimental methodology, the absolute reference value may be derived by scientists that construct the instrument, in which case they will need to set their own CRM. Thereby, they will need to report their experimental values subject to a traceable reference paper that defines their internal standard.

Experiments that employ absolute reference values are typically extractable from scientific literature, although their reliability may be questionable unless their absolute reference value can be identified and sourced. This is often not straightforward because absolute reference values tend to be listed in quite obscure literature; although they are at least more likely to be published than relative reference values.

*Relative reference data* tend to be employed when performing measurements on custom experimental instrumentation to normalize the data or correct the data for known artefacts.

For instance, one may need to normalize experimental results between different laboratories that use custom fabrication methods so that the results are globally comparable. A well‐known example involves the concerted measurement of device performance data on two solar cells that have been fabricated in the same experimental batch, one of which contains a new prospective light‐harvesting chromophore (the sample of interest), whose device performance metrics are normalized against those of the other solar cell that contains a well‐known light‐harvesting chromophore (a reference material). Note that the device performance of the internal reference solar cell in this case can, in turn, be related back to absolute literature values from an external reference [54].

Another common type of relative reference measurement in materials science concerns background data. Thereby, one often needs to subtract one or more types of background data from sample data, as a distinct data‐processing step. This background‐subtraction step removes signals that have been detected from the physical or chemical environment which surrounds the sample, thereby contaminating the sample data. Types of sample environment include detected signal from: the intrinsic characteristics of the measuring instrument (i.e., the empty instrument under a given set of experimental conditions); the sample container (e.g., a cylindrical can that holds a powder sample); ancillary equipment (e.g., a cryostat that cools the sample); and a material matrix in which a sample has been incorporated to enable a measurement (e.g., the sample diluted into a pressed solid pellet). A complex experimental setup may well exhibit all of these types of background data; in such an event, relative reference data need to be collected on the isolated background environment, for example, a spectrum of an empty instrument that houses the empty sample container, a sample of the raw matrix materials in which the sample will be embedded, and the installed ancillary equipment.

These background data signatures will then be removed from the sample data once they have been collected.

Both types of instrument‐specific relative‐reference data are rarely published; instead, only the normalized or corrected data are presented. This is suitable insofar as the normalized or corrected data do not need to be unpacked, but this lack of traceability back to the raw data leaves the efficacy of the salient experimental data vulnerable to challenge.

##### 4.5.2.6 Deliberately Non‐Published Data

A scientist may also choose not to publish a set of data on strategic grounds. For example, certain data may be withheld from public view to prevent unwanted political reactions, or to protect national security. Other data may be withheld from publication if this would reveal scientific results or technical ‘know‐how’ that researchers wish to exploit, which could be copied by a competitor in a timely fashion; essentially, the researchers are withholding data strategically to gain competitive advantage. A company may take a broader view of competitive advantage by protecting all its ‘know‐how’ as a trade secret. Given that strategic reasons lie behind the non‐disclosure of this type of ‘dark data’, such data are likely to remain latent, at least while they hold considerable value.

##### 4.5.2.7 Incomplete, Uncertain or Ambiguous Data

A scientist may also wish to withhold data from publication if the results are incomplete, uncertain or ambiguous. Nonetheless, a range of statistical imputation methods have been developed to overcome the pervasive issue of incomplete data in materials science [116], even though the population of imputed data into empty records of datasets can introduce imputation‐induced errors (IIEs) during this process. Codebases that minimize IIEs have been developed to aid this data‐cleaning process for materials science; one notable example being *MatImpute* [117]. Meanwhile, the combined use of multiple‐imputation methods to accommodate data‐scarcity issues has proven effective; an interesting use case being the prediction of battery lifetimes using multiple‐imputation chained equations [118]. Further developments in imputation methods will hopefully help unlock more ‘dark data’ within the energy sector.

The innate quality of experimental data that are available for a given study also needs to be considered, within the context of their typical data scarcity. Approaches to combat data scarcity to solve materials‐science problems include combining models and datasets via a mixture‐of‐experts framework [119] as well as data augmentation, feature engineering, transfer learning, and active learning [120].

Data‐quality criteria can be formulated to deal with uncertain and ambiguous data. Setting criteria that establish what is fit for purpose to solve a given problem is a particularly helpful approach; thereby, the use of confidence intervals and sensitivity analysis is valuable [121]. The trustworthiness of the source data is also an increasing part of this data‐quality task, given the recent rise in AI‐generated data that can emulate experimental data in materials science so well that they are indistinguishable by a human [122].

Further improvement of these statistical methods will hopefully foster enough trust that the scientific research community will consider publishing more of this type of data. This review suggests that the creation of a new type of metadata that describes associated data to a specified level of data efficacy may help to incentivize this process, as it would protect the scientist with a statistically informed caveat as to the reliability of the source data.

Unless a major disruptive change to the *status quo* is realized in how scientists deal with dark data and their associated metadata, irrecoverable practical issues in dark data and data reuse will persist. Taking the field of batteries as just one example, current practical limitations in locating dark data include: non‐reporting of experimental data that describe failed chemical compositions, synthesis‐window boundaries, non‐optimal electrolytes, unstable battery‐cell cycling; while the reuse of dark data in this field is inhibited by high levels of uncertainty and data‐efficacy issues, such as a lack of data provenance, standardized units, controlled vocabularies and interoperable APIs.

#### 4.5.3 Opportunities to Unlock ‘Dark Data’ in Materials Science From Experimental Laboratories

While the likes of giant software companies are harvesting all knowledge from the internet to create knowledge‐based AI‐technological applications such as ChatGPT, [1] grey data in materials science is not necessarily likely to lighten as much as the AI industry would wish, without the deliberate and strategic intervention of grey‐data‐extraction efforts. This is partly because processed data present with highly specialized vernacular and are often labelled with custom abbreviations that render impossible the forging of any direct link between chemical names and their properties, such that finding patterns from structure‐property relationships is intractable. Meanwhile, raw data are so often uninterpretable because they have not been stored with associated metadata that critically define them. Additionally, raw data may be deemed intractable because they have been found to be unreproducible, or they have not been standardized at source, for example, measured relative to a benchmark reference (see discussion on reference data in Section 4.5.1.5). Such data heterogeneity seriously hinders the reuse of experimental dark data even when they have been carefully catalogued. Furthermore, a massive volume of raw data remains offline; as such, it cannot even be found by internet search engines. Overall, experimental data need to be stored in a findable, accessible, interoperable, or reusable (FAIR) fashion [123].

Instead of focusing on legacy data, managing the forward capture of ‘dark data’ directly from the experimental laboratory offers a major opportunity for materials science. Thereby, three rich troves of ‘dark data’ that could be extracted are worth special mention as they tend to contain massive volumes of orphan data, negative data, positive data and unpublished data: laboratory notebooks, high‐throughput experiments (HTE) and digital twins (especially their real‐time sensor data).

##### 4.5.3.1 Laboratory Notebooks

Logs of experiments performed in a laboratory are typically recorded using either hand‐written or electronic laboratory notebooks (ELNs). Hand‐written notes are, of course, the more traditional method of capturing metadata during an experiment. Notebooks of this type have not historically tended to be scanned into electronic media. This is especially true of old logbooks created in academia, whereby myriad hand‐written logbooks have left universities with their authors, largely comprising PhD students and postdoctoral staff; given the fixed‐term nature of such researchers and the lack of a corporate need to maintain all scientific information that is created within a given laboratory, particularly once it has been published. These notebooks have therefore become strewn across the world and are unlikely to become recoverable unless the work in these logbooks or their authors subsequently become famous. As such, myriad academic logbooks have been lost to time. Nonetheless, master's and PhD theses have been lodged and stored in many university libraries, which capture structured content of laboratory notebooks. This content can be scanned from these theses, although its form predominantly consists of positive scientific results rather than null or negative results, which people generally wish not to report, especially in examinable tomes. So, there will be a strong positive bias in what scientific knowledge can be recovered. Industrial organizations have fared better in maintaining legacy experimental metadata and data, having captured old recordings of experiments on microfiche; and many legacy handwritten logbooks from companies have since been manually scanned into electronic files to manage their industrial ‘know‐how’. Notwithstanding the aforementioned difficulties in capturing legacy metadata and data from laboratory notebooks or old file formats, our increasingly digital world presents a golden opportunity to fix this issue in going forward.

The increasing trend toward scientists capturing data and metadata from the experimental laboratory in digital form offers a valuable opportunity for translating this information into materials databases, pending that it is collected within a suitable framework, for example, an ELN. There are now so many open‐source ELN tools for materials science that there exists an ELN Finder tool for this scientific domain [124]. The scientific contents of ELN data are particularly diverse as ELNs record a plethora of detailed procedures, observations, logs, notes, results and findings that a scientist has intuited about a wide variety of experiments and measurements. Moreover, ELN data and metadata may be recorded in formats that range from highly regulated to completely freestyle, using accepted terminologies, units, ontological mappings, descriptions and so forth, or custom labels, abbreviations, classifications, sketches and correlated mappings of data. Laboratory data are not typically logged anywhere else, unless they are part of the very small subset that are used in a publication. They frequently contain experimental ‘know how’ that may have ‘trade secret’ value, that gives an organization competitive advantage. This poses certain security considerations on these types of data. They also contain a wealth of negative or orphan data since many experiments, however well designed, do not afford positive or intended results, respectively.

It is important to remember that laboratory notebooks capture information about metadata and data at the start of the data‐curation process. Therefore, they represent a particularly important type of ‘dark data’ as all downstream data and metadata depend on them. Indeed, the full value of AI‐ready datasets would be automatically realized if the full contents of an ELN and their acquisition data for a given dataset were captured and integrated into an experimental workflow that tracks the entire *digital thread* of data and metadata along a fully digitalized supply chain, from data source to sink, that is, to cohesively generate machine‐readable datasets. Ideally, this digital thread would carry the option of a feedback loop where it entails experimental measurements that are to be acquired in multiplicate or if a series of similar experiments are to be collected in a sequence. This feedback loop would provide a means by which experiments could be adapted and optimized if needed, or correlated data could be amassed where relevant. Such digital threads are gradually being incorporated into experiments as they become more automated, particularly with regard to HTEs and SDLs.

##### 4.5.3.2 High‐Throughput Experiments (HTEs)

HTEs tend to be conducted on a focused set of chemicals (*x*) which are systematically exposed to a pre‐defined range of conditions (*y*) in a matrix style of enquiries. They are subjected to statistical (typically Bayesian) logic that optimizes a sequence of experimental measurements to prevent an entirely exhaustive row‐by‐row raster of (*x*,*y*)‐data unless it is actually necessary. HTEs are typically pre‐set with a specific objective that meets an overarching materials‐science goal. HTE datasets thus cover a rich, but narrowly focused, area of chemical space. Given that HTEs seek a specific objective as the salient scientific result, only one or a few (*x*,*y*)‐data points transpire to be of particular interest, leaving the surrounding matrix of (*x*,*y*)‐data somewhat redundant. Yet, such (*x*,*y*)‐datasets offer a plethora of negative (null) data and positive but unwanted (orphan) data by virtue of the experimental design: an HTE is geared to reject all data unless they meet a pre‐defined objective that informs a given scientific hypothesis. Thus, HTE datasets represent a trove of negative and orphan ‘dark data’; whose digitally orchestrated form of creation is eminently poised for their automatic labeling with cognate metadata and their subsequent storage in a highly structured open‐source data repository. Furthermore, if the algorithm used to optimize the sequence of experimental measurements is stored with the HTE dataset, together with its tracked (*x*,*y*)‐measurement route, then one can presumably estimate well the values of any (*x*,*y*) sampling region where data were not collected by statistical imputation.

HTEs are increasingly being performed in materials science, including the energy sector; some prominent examples from the fields of batteries, catalysis and photovoltaics are now discussed.

The field of batteries is fueled by electrochemistry. Therein, an HTE has identified an electrolyte additive for aqueous zinc metal batteries that could deliver high Coulombic efficiency [125]. Other experimental platforms, set up for the high‐throughput synthesis of thin films, have uncovered multiple materials‐substitution strategies for battery applications: for example, magnesium can be used to substitute for iron in a LiFe_1−y_Mg_y_PO_4_ battery precursor mixture to afford improved battery performance [126], a new synthesis route has been discovered for producing pure phase sodium‐ion cathode materials by systematically exploring the Na−Fe−Mn−O compositional space with HTEs [127]. Materials‐substitution strategies have been highlighted by the EU Critical Raw Materials Act [72] as a prospective solution to overcome current difficulties in accessing critical raw materials (one of which is lithium).

HTEs in the field of catalysis have demonstrated manifold success with examples including: the discovery of high‐performing material compositions of Fe_x_Co_y_La_z_‐containing electrocatalysts for water splitting, whose catalytic stability and mass activity are superior to that of RuO_2_ [128], realizing green‐hydrogen production using photocatalysts, where researchers have shown how the photocatalytic activity of initial formulations could be improved by six times, via a robot‐assisted HTE [96], alternatively, realizing green‐hydrogen production using electrocatalysts from the Mn–Sb–Sn–Ti–O system, whose chemical composition has been optimized for low Mn concentration to improve stability [129]. Incidentally, Mn is a critical raw material because it is a key material ingredient in batteries for electric vehicles (EVs) [130], so lowering the need for Mn content in this electrocatalysis application is also helpful to this end.

HTE technology has also extended to the field of photovoltaics. For example, an HTE setup has been built to optimize the open‐circuit voltage and fill factor of solar cells for organic photovoltaic (OPV) applications [131]. Thereby, the electron donor:acceptor ratio of two polymer blends was systematically varied during automated measurements that track the concerted data collection of UV/vis absorption spectra, current‐voltage curves, and microsecond transient‐absorption kinetics; the resulting charge densities were used to optimize the Langevin reduction factor, that is, optimize the extent by which charge is extracted over its competing tendency for charge recombination. In other work, another type of HTE set up for photovoltaic applications [132] uncovered four lead‐free layered perovskites, A_3_B_2_Br_9_, where A is Cs or Rb and B is Bi or Sb, and a related multi‐site lead‐free series of Cs_3_(Bi_1‐x_Sb_x_)_2_(I_1‐x_Br_x_)_9_ chemicals; their band gaps ranged from 1.2–2.4 eV, giving them potential as light‐harvesters for perovskite solar cells.

The above examples of HTEs collectively demonstrate the ability to automate all parts of a ‘design‐to‐device’ supply chain to run systematic studies on molecular design, chemical synthesis, materials characterization, device fabrication and testing. In principle, the extent of automation on each type of HTE could be increased to the ultimate point that all operational components of a ‘design‐to‐device’ supply chain are connected in a contiguous fashion for a given case study. Once such a supply chain is fully integrated with closed‐loop positive feedback functionality, it could operate autonomously by exploiting the latest robotic and AI technologies. Such an HTE would share more in common with an SDL that was discussed in Section 4.3.

However, there are important distinctions between HTEs and SDLs. In particular, HTEs are more versatile and accessible than SDLs in that they can be set up readily as ‘pop‐up labs’ to conduct experiments of a limited duration to test many material samples against a particular set of hypotheses; so, they can deliver measurements rapidly in a high‐throughput manner that facilitates good cost efficiency. Meanwhile, the fully autonomous, closed‐loop nature of SDLs produces high‐throughput measurements using decision‐making with adaptive strategies that consult a large number of interdependent variables to optimize the experimental conditions. This affords SDLs with superior quality control, higher levels of operational efficiency (notwithstanding their need for technical maintenance), and greater capabilities for setting up highly complex experiments or manufacturing processes that typically mandate very high levels of technical specification and performance criteria.

Notwithstanding all these state‐of‐the‐art scientific and technical advances, not even the best combination of experimental and computational frameworks can fully simulate the behavior of myriad materials once they have been incorporated into their industrial products and set to operate in their real‐world environment. This knowledge gap is common, even when one has managed to quantify all individual relationships between a material and: its structure and cognate properties at all scales, its compatibility with the device physics that is associated with the intended industrial product, its device fabrication and manufacturing processes, its chemical stability once incorporated into a device, the processing conditions that it must withstand to be incorporated into a product. The only way to overcome this piecemeal understanding of a material is to perform *in operando* experiments on the industrial product within which the material is contained. Such experiments tend to be very costly, and the measurements sought are often not even tractable for various practical reasons. Fortunately, global efforts toward a worldwide digital transition have stimulated the technological development of *digital twins*.

##### 4.5.3.3 Digital Twins

The basis of a digital twin (DT) is the virtual representation of a physically operating system that can simulate its real‐time behavior via dynamic data communication between the virtual and physical system [133]. This concept is thought to originate from the Mirrored Spaces Model [134]. A more detailed definition of a DT is not yet agreed universally [135], although most now seem to accept that a DT should be able to dynamically update its virtual configuration with a continuous feed of experimental data that it receives from the physical system and vice versa; that is, a DT should carry a bidirectional data flow [133]. Some argue that the form of a virtual representation must digitally replicate the physical system; while more modern definitions tend to accept a DT to have a virtual representation whose digital form reproduces the *behavior* of the physical system without an added mandate to emulate its physical form [135].

Either way, the digital and physical real‐time data synchronization of a DT enables real‐time system simulations. The continuously interfacing data‐feedback loop between the virtual‐physical system in a DT allows one to observe, predict and optimize system performance throughout its entire life cycle [135]. This is important because it means that DTs can be used to make operational decisions that produce gains in the efficiency and quality control of a system, making DTs incredibly valuable for industrial growth.

It is therefore not so surprising that DTs originated from the engineering sector, where they were first applied to high‐value machines whose reliable operation was of paramount importance; a high‐profile example being the DT that was constructed by NASA to study the Apollo missions [136]. The wider aerospace industry followed suit in adopting DTs so extensively that they are now used to prototype, test, or optimize the performance of all sorts of machines and processes, from jet engines to entire aircraft, to their manufacturing processes and life‐cycle analysis [137, 138, 139].

This highly successful adoption of DTs in the aerospace industry has motivated other sectors to develop their own DT technologies, which has been spurred on by the perfect storm of concomitant developments in computer‐aided design (CAD) [140], the Internet of Things (IoT) [141], probabilistic simulation [142], statistics (including machine learning and data science) [143, 144], and the evolution of software‐driven product lifecycle management [134] (including life‐cycle analysis methods) [145].

The collective power of these developments has enabled a vast expansion in the scope of physical systems to which the DT concept can be applied. Thereby, DTs are now being constructed by many industries to help them make decisions about the operational performance of objects that span devices, materials, machines and instruments (or components thereof), as well as processes that cover all sorts of synthesis, fabrication, manufacturing or evaluation diagnostics. These industrial applications include myriad aspects of two overlapping industries: materials science and the energy sector.

Thereby, DTs are deemed to hold particularly high prospects for enabling the sustainable provision of materials, and sustainable energy resources, for each nation while protecting our planetary resources. This is because a life‐cycle analysis (LCA) of materials is important for realizing sustainable materials and energy, with reuse and recycling options being critical to function; while DTs possess an innate data circularity via their continuous data‐feedback loops which map abstractly, but directly, to the critical needs of a circular economy. Note that the operational provision of sustainable materials and energy also bears high strategic relevance to international initiatives such as the EU Critical Raw Materials Act [72] and the EU Net Zero Industry Act [146].

The emergence of DT applications to materials for sustainable energy will now be discussed. Cases are taken from the fields of batteries and photovoltaics given that clear momentum toward digital twins is evident in these sectors; while a few interesting standalone examples from the thermoelectric [147] and semiconductor [148] materials arenas are noted herewith for the interested reader.

An obvious application of batteries is in electric vehicles (EVs). DTs of EV powertrains exist [149, 150], although an entire EV is naturally far more complicated than a battery. Fortunately, a battery is a very modular component of an EV. So, a much simpler and far more accessible DT can be created purely from a battery, as long as its generated data can be communicated to an EV and vice versa. In principle, such data communication could be enabled using a battery management system that employs an End‐Edge‐Cloud network which links a dynamic two‐way data feed between the processing unit of an on‐board end application (an EV), through edge computing nodes and cloud computing, to and from the battery DT [151]. However, access to DTs of EVs tends to be heavily protected by corporations because they are high‐value assets. Corporate concerns include data leakage from such DTs that would cede company know‐how about their specific EVs as well as compromise their cyber‐security.

Notwithstanding, the fully engineered DT of an EV could be protected by creating a DT specifically about an EV‐styled battery, with its findings being transferable to EV technology. This approach is also far simpler and more accessible than dealing with a DT of an entire EV; albeit, the DT of a battery for EV applications may need to host boundary conditions that constrain certain operational parameters so that its scope remains relevant to EV applications. The DT of a battery would also benefit strongly from the use of a battery management system, such as the Cyber‐Hierarchy and Intelligence Network (CHAIN) [152], that incorporates the multi‐scale and multi‐temporal value chain of the materials‐engineering process for a battery: that is, the atomic structure and morphology of various battery material components, fabrication and manufacturing aspects of battery electrodes and electrolytes, through to the cloud‐based modeling of batteries.

These complex requirements of a DT for a battery pose substantial R&D challenges. Nonetheless, the field of batteries is certainly targeting the application of DTs. Based on the academic literature, the current state‐of‐the‐art in battery technology is perhaps better described as having reached that of a *digital model* [153, 154, 155], even though the ultimate goal is to realize a DT. Liu et al. [153] describe how a multi‐scale materials‐characterization framework needs to be incorporated into digital models for battery materials to step toward this ultimate goal, while Kim et al. [154] explain how this multi‐scale modeling needs to be synergized with AI and dynamic simulation techniques to develop the foundations of a DT for batteries. Meanwhile, Zhang et al. [155] have managed to link a virtual and physical model of a battery and manually share data between these two models to help optimize them in a stepwise fashion; that is, realizing a two‐way data communication, even though these models lack the automated, dynamic, continuous bidirectional flow of data that would manifest in a DT. The capture and movement of data across this virtual‐physical interface is also encouraging because it affords a *digital thread*, that is, a full history of all data and metadata in a digital system captured across time and space.

The development of DT capabilities in the field of PVs appears to be in a similar position to that of the battery industry. Thereby, the engineering sector dominates current technological advances in PV‐based DTs, where their key operational roles in solar‐power plants have been demonstrated with good effect. A case in point is the international initiative, Solar Decathlon AFRICA, where a DT framework has been established across a suite of building‐adapted photovoltaic (BAPV) installations in a ‘living lab’ solar‐power plant in Ben Guerir, Morocco [156]. This solar village was set up as a platform for experimental research in solar energy, and has now become known as Green Energy Park [157]. Kaitouni et al. have shown that this DT framework can successfully optimize the spatiotemporal energy performance of a distributed solar‐power plant in an agile manner [156]. Thereby, the DT framework managed to run diagnostic tests that could detect, diagnose, and monitor technical faults and anticipate operational maintenance needs. The DT framework achieved this by synergizing its dynamic physical component (solar‐cell plant operation) with its virtual twin that comprised a range of analytical and statistical models that predict PV power output. The latter included support for a range of machine‐learning models that could readily be trained on large sets of operational data that are produced at the plant from which they could learn; and thence predict data anomalies (e.g., representing a localized fault in a solar panel) or unusual signal drift (e.g., from the progressive performance degradation in an aging PV panel). Real‐time monitoring and sensing by this ground‐based DT framework was supported by aerial surveillance that employed drones, whose plan view of the solar panels enabled the cause of power degradation in certain PV modules to be diagnosed using image‐recognition software as its virtual component. Typical causes include environmental conditions, such as Saharan dust or bird droppings, that could block PV panels from capturing sunlight [156, 158]. Demonstrations of DT‐enabled solar‐power plants have assisted their operational staff in developing improved data‐driven decision‐making to optimize power output [159].

Notwithstanding, the distributed nature of DT frameworks within solar‐cell plants makes them vulnerable to cyber threats, so risks to data‐leakage that could compromise in data security and privacy are a key concern that needs to be overcome [159]. Another key issue is the extent to which machine‐learning models for PV‐based DTs are transferable across solar‐cell panels or entire plants; noting that a set of data from one solar‐cell panel may hold a different configuration to another or be exposed to disparate environmental conditions. Zhao developed a transformer‐based machine‐learning model that can be domain‐adapted to improve transferability of a virtual model within a PV‐based DT [160]. Subject to ensuring their adequate transferability, DTs of solar panels could also be employed to help design new solar farms [161, 162].

While these PV‐based advances in DT technologies appear to be a long way from materials science, one can holistically learn from these engineering successes to help develop the materials and manufacturing aspects of the PV value chain. For example, the massive volumes of data being accrued on these solar‐panel operations could be useful as a benchmark when developing a DT for solar‐cell manufacturing. Moreover, the stress‐testing of machine‐learning‐model architectures within DTs that aim to improve solar‐panel operations will indirectly facilitate the development of DTs for the PV‐based materials and manufacturing sector. This is because they carry the same type of PV data; for example, Zhao has produced a bespoke machine‐learning pipeline that has been designed to handle time‐series PV data [160].

The current state‐of‐the‐art in the PV materials and manufacturing sector could be considered akin to the aforementioned state of battery technology that is better described as having reached that of a *digital model*. For example, a digital model has been showcased in the roll‐to‐roll manufacture of solar cells [163], which takes the form of a high‐throughput experiment whereby 11 800 organic PV devices were fabricated, characterized, and tested within a 24 h period. The report notes that sensors are embedded with closed‐loop capabilities, with a cloud connection. This setup is sophisticated; albeit, it does not yet appear to meet the two‐way dynamic physical‐virtual criterion of the herein reviewed digital‐twin definition. While digital‐twin developments appear less mature further up in the materials ‘design‐to‐device’ supply chain, the PV materials community certainly aspires to develop DTs for solar‐cell technologies [132, 164]. Thereby, high‐throughput experiments that synthesized and tested of 75 thin‐film perovskite material compositions for solar cells are a significant step in this direction [132], while others detail the materials‐centered potential that could be afforded by PV digital twin technology [164]. From an energy‐sustainable perspective, a PV‐based digital twin that covers the full multi‐scale, multi‐temporal value chain of PV materials will hopefully help encapsulate the life‐cycle‐analysis opportunities of this energy sector.

Overall, the development of digital twins for data‐driven materials science appears to be nascent, while their potential for unlocking ‘dark data’ is stark. Thereby, digital twins offer materials science the prospect of a vast, continuous feed of real‐world, real‐time data from which researchers will be able to apply to solve key materials and manufacturing problems in the energy sector.

Accordingly, such digital twins stand to offer some of the highest value data of all. So, it is not surprising that industry has invested in them ahead of academia and that they are emanating from the engineering sector into materials science, while most materials‐centered developments go in reverse. In principle, collaborations between industry and academia are possible, but the high stakes of many industrial data from digital twins render some serious considerations about intellectual property and data security. Such data is unlikely to become open access. Yet, it may be possible to forge industry‐academic collaborations that share such data in materials science, while protecting the high‐value data assets via some sort of federated data access scheme. These types of schemes have shown good dividends in other sectors, such as medicine [111].

## 5 Conclusions

Materials science has witnessed a coming‐of‐age in automated, high‐throughput data extraction from scientific literature over the last decade. It has become possible to auto‐generate a customized experimental materials database and use it to realize a domain‐specific materials goal with a data‐science strategy. Thereby, six case studies that employ distinct auto‐generated experimental materials databases have herein illustrated how six types of data enquiry can be fielded by data‐driven methods to serve the energy sector. The sixth case study combined the use of an auto‐generated materials dataset with a language model that had been trained on this materials domain in order to classify some of its data records.

This data‐driven approach stems from an almost boundless set of future opportunities, not only for auto‐generated materials datasets, but also for newer representations of knowledge that align with AI developments. Thereby, the world is currently witnessing an evolution from domain‐specific datasets to models, to decision‐making workflows, to solve materials‐science challenges. These newer, integrative knowledge and AI models need to be underpinned by richer, high‐quality streams of data. This is escalating a major challenge in data availability and energy costs for materials science. This review has showcased some possible solutions to this challenge, while maintaining its focus on employing experimental data. To this end, the use of electronic laboratory notebooks (ELNs), high‐throughput experiments (HTEs), and digital twins (DTs) offers particularly good opportunities in going forward.

While computational data are outside the scope of this review, it is helpful to add a brief comparative between computational and experimental data at this juncture. Figure 16 illustrates some key points to this end. Experimental data are very heterogeneous in nature because they tend to derive from myriad measurements of material data that were collected by all sorts of individuals across many laboratories around the world under diverse sample environments. The collated set of such data tends to be patchy across *chemical space*, which is defined here as the universe of all possible chemical compounds that could ever exist. The distribution of black dots in the yellow elliptical region of Figure 16 represents this patchiness of experimental data across three dimensions of chemical space that span chemicals as a function of: the number of atoms per molecule that they could possess, the proportion of all possible chemical elements that they could contain, and the number of viable data records that they would collectively afford. This heterogeneous distribution contrasts with the almost complete coverage of data within the rounded rectangular blue region of Figure 16 that depicts how electronic‐structure calculations can raster across all possible chemical space within a given region, subject to these calculations being able to model constitutional atoms up to a certain atomic number and up to a certain number of atoms per atomic or molecular structure. In principle, such calculations could produce a never‐ending supply of data records pending the availability of supercomputing resources and human expertise. This blue computational region could of course be massively expanded by allowing generative AI to propose more chemical compounds, although the scope of Figure 16 has been restricted to deterministic calculations to ensure that the chemical space represented is at least structurally viable. Overall, Figure 16 shows that experimental data can span a wider range of chemical space than computational data, albeit with much more patchy coverage.

**FIGURE 16:**
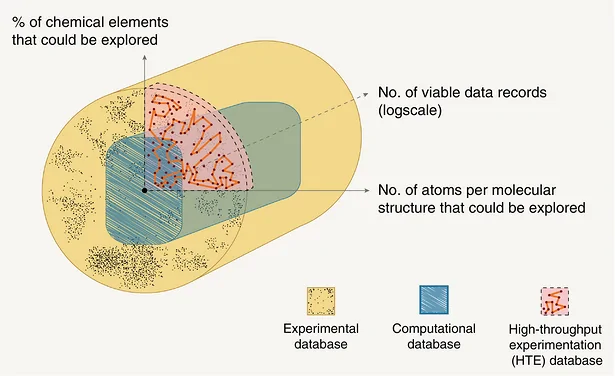
Shape of the explorable chemical space. It is mapped across three axes: percentage of all possible chemical elements, number of atoms per molecular structure, and number of viable data records (log scale). Experimental data (yellow) span the widest region of chemical space, albeit data coverage is patchy (cf. black points). Comparative data ranges and coverage of chemical space are given for computational data (blue) and HTE data (pink).

An interesting middle ground is offered by HTEs where a very rich set of data is acquired over a small, but highly concentrated region of chemical space. Thereby, HTE data are automatically collected using a raster method that typically tracks a Bayesian optimization algorithm to prioritize the acquisition of data at points in chemical space that hold the greatest relevance to a target goal, for example, a point in chemical space that conforms to a lead candidate in a data‐driven material‐discovery workflow. The red segment in Figure 16 shows how this HTE process manifests in chemical space. These more targeted types of experimental measurements offer a faster track toward increasing the value chain of data‐driven materials science. The automated nature of HTEs also stands to mitigate various types of experimental uncertainties that are caused by human error. The previous section showed that this value chain of data‐driven materials science can be further increased by employing digital twin technologies, where the focus is on just one point of chemical space (Figure 15). Examples were given for dynamically optimizing the operational performance of silicon solar cells as well as batteries. Looking ahead, there appears to be a massive, but largely unmet, opportunity to develop DTs to optimize advanced manufacturing processes for sustainable energy applications.

Further developments in AI models that employ a continuous feedback loop to self‐improve, such as those incorporated in DT technologies, could help offset a common limitation of ML models that make predictions about materials; whereby, the model performs well for in‐sample predictions but performs poorly when applied to out‐of‐sample predictions.

More generally, the development of AI models that relate more closely to real‐world materials problems is likely crucial to their long‐standing utility. Such AI models need to be trained on experimental data, at least in part, so that they can reliably predict the characteristics of a material which are critical to their function and yet are currently beyond the capabilities of high‐throughput computational calculations. For example, materials‐characterization data can describe interfacial structure or surface reconstruction, identify the nature of dopants or defects in materials and quantify their levels, and understand the influence of chemical additives on the operation of a device. The ability to relate these sorts of characteristics across multiple scales, from molecular to macroscopic levels, is also critical to function. To all these ends, experimental data can provide unique insights. The energy sector is a case in point. For example, interfacial structure, chemical additives, dopants and defects in materials can all influence the performance of a battery [165, 166], while the surface structure of a material can govern how a heterogeneous photocatalyst can accelerate water splitting [167].

Ultimately, a data‐driven ‘design‐to‐device’ approach to materials science for sustainable energy applications which combines the assets of experimental and computational data could offer a good way forward. Such multi‐fidelity AI models are still realizing their potential, as are multi‐modal AI models and multi‐scale approaches.

The fast‐moving nature of AI will inevitably produce many more innovations in this field that we cannot even begin to foresee at this juncture. Nonetheless, these final recommendations will hopefully help steer the research community toward next steps that can maximize the application of AI to data‐driven materials science for energy sustainable applications. Thereby, we can act collectively as a force for good to orchestrate materials science in a way that safeguards the future of our planet.

## Conflicts of Interest

The author declares no conflicts of interest.

## Data Availability

The data that support the findings of this study are available from the corresponding author upon reasonable request.
